# Quenching Single-Fluorophore Systems and the Emergence of Nonlinear Stern–Volmer Plots

**DOI:** 10.1021/acsomega.5c13199

**Published:** 2026-02-09

**Authors:** Ronen Zangi

**Affiliations:** † Department of Organic Chemistry I, 16402 University of the Basque Country UPV/EHU, 20018 Donostia-San Sebastián, Spain; ‡ Donostia International Physics Center (DIPC), 20018 Donostia-San Sebastián, Spain; § IKERBASQUE, Basque Foundation for Science, 48009 Bilbao, Spain

## Abstract

Reduction in fluorescence intensity upon addition of quencher molecules is often quantified by the Stern–Volmer equation. Central to the underlying model is the formation of an adduct between quencher and excited state (dynamic quenching), or ground-state (static quenching), fluorophore at steady-state conditions. Assuming a thermodynamic behavior, that is, a system with large numbers of fluorophore and quencher molecules, the resulting dependency of the ratio between fluorescence intensities, with and without quencher, on quencher’s concentration is linear. Yet, alongside abundance reports confirming this linear behavior, numerous observations indicate the dependency can also be nonlinear with either upward or downward curvature. By maintaining the same physical mechanisms for quenching, we derive in this paper an alternative equation to describe fluorescence quenching. Here, however, we assume a local equilibrium (steady-state) between a single fluorophore and a finite number of surrounding quencher molecules, effectively partitioning the (macroscopic) system into many noninteracting small subsystems. Depending on the fluorophore’s properties, the association’s strength, and conditions, the resulting behavior exhibits linear dependencies, upward curvatures, or downward curvatures. More specifically, the relation reads, $I_{{}^{\circ}}/I=1+ZK{\left[Q\right]}_{T}/\left(1+\left(1-Z\right)K{\left[Q\right]}_{T}\right)$ , where *K* is a steady-state equilibrium constant for complex formation and [Q]__*T*__ is the total concentration of quencher in the small subsystem. The dimensionless parameter Z has different expressions for dynamic and static mechanisms. In the former, it is a ratio between the maximum rate of quenching and the rate of fluorophore excitation, whereas in the latter, it is a function of the fraction of excited fluorophore. Intriguingly, this relation applies also for systems with exciplex emissions. We tested the validity of this model on 151 experimental fluorescence quenching plots, taken from the literature, operated by dynamic, static, and combined mechanisms. The results of the fitting are excellent with an average correlation coefficient of 0.9985.

## Graphical abstract

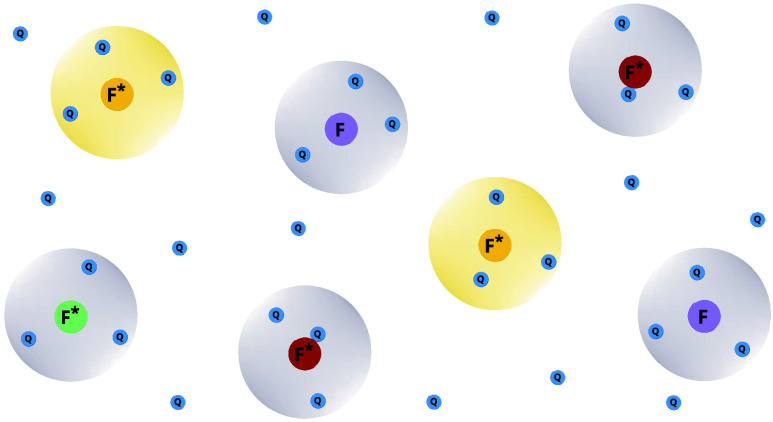

## Introduction

Consider a system with fixed volume, *V*, temperature, *T*, and total number of fluorophore molecules, *N*_F_^total^. In this system, a fluorophore molecule in the ground state (F) can be electronically excited (F*) by absorbing a photon with frequency ν. If the light source applied to the system emits photons with constant intensity, the excitation process of fluorophore molecules can be described by first-order kinetics

1 $$F\overset{+h\nu }{\underset{k_{a}}{\to }}F^{*},\mathrm{rate}=\left\langle \partial c_{F*}/\partial t\right\rangle =k_{a}\left\langle c_{F}\right\rangle$$

where *h* is Planck’s constant, *k*_a_ is the rate constant of photon’s absorption, *c*_F_ = *N*_F_/*V* is the concentration of ground-state fluorophore, and angle brackets indicate an average over measurement time. The excited fluorophore can go back to its ground-state by two different routes. The first is by fluorescence

2 $$F^{*}\overset{-h\nu^{\prime }}{\underset{k_{f}}{\to }}F,\mathrm{rate}=-\left\langle \partial c_{F*}/\partial t\right\rangle =k_{f}\left\langle c_{F*}\right\rangle$$

where *k*_*f*_ is the fluorescence rate constant and ν′ the emission frequency. The second route is via nonradiative relaxations

3 $$F^{*}\overset{k_{nr}}{\to }F,\mathrm{rate}=-\left\langle \partial c_{F*}/\partial t\right\rangle =k_{nr}\left\langle c_{F*}\right\rangle$$

where *k*_nr_ is an effective rate constant representing all first-order nonradiative relaxation modes.

For a system subject to reactions 1–3 under continuous irradiation of photons, it is customary to assume a steady-state condition. When applied to the population of excited fluorophore, ⟨∂*c*_F*_/∂*t*⟩ = 0, we get,

4 $$\left\langle c_{F*}\right\rangle =\frac{k_{a}}{k_{f}+k_{nr}}\left\langle c_{F}\right\rangle$$

The rate of emitted photons by these excited fluorophores (i.e., the rate of fluorescence described in eq 2) is linearly proportional to the fluorescence’s intensity observed. Thus, in the absence of a quencher, the fluorescence intensity, $I_{{}^{\circ}}$ ,

5 $$I_{{}^{\circ}}\propto \frac{k_{f}k_{a}}{k_{f}+k_{\mathrm{nr}}}\left\langle c_{F}\right\rangle$$

Now, if we add a molecule, Q, to the system that can bind the fluorophore, the fluorescence intensity can be reduced (quenched). This fluorescence quenching can take place either by a dynamic or static mechanism.

### Dynamic Quenching

In this mechanism, the decrease in fluorescence intensity is a result of the association between the excited-state fluorophore and quencher, allowing the former to relax back to its electronic ground-state by another nonradiative pathway. Given the excitation in eq 1, the subsequent step is then the formation of the excited bound complex, (FQ)*

6 $$F^{*}+Q\overset{k_{b}}{\underset{k_{d}}{⇌}}{\left(\mathrm{FQ}\right)}^{*},\mathrm{rate}=-\left\langle \frac{\partial c_{F*}}{\partial t}\right\rangle =\left\langle \frac{\partial c_{\left(\mathrm{FO}\right)*}}{\partial t}\right\rangle =k_{b}\left\langle c_{F*}\right\rangle \left\langle c_{Q}\right\rangle -k_{d}\left\langle c_{\left(\mathrm{FQ}\right)*}\right\rangle$$

where *k*_*b*_ and *k*_*d*_ are the binding (second-order) and dissociation (first-order) rate constants, respectively. The dynamic pathway proceeds with a step, or several steps, in which the excited bound complex dissociates into the fluorophore and quencher, both in their ground electronic state

7 $${\left(\mathrm{FQ}\right)}^{*}\overset{k_{i}}{\to }F+Q,\mathrm{rate}=-\left\langle \frac{\partial c_{\left(\mathrm{FO}\right)*}}{\partial t}\right\rangle =k_{i}\left\langle c_{\left(\mathrm{FQ}\right)*}\right\rangle$$

Using eq 6 and eq 7, we can apply a steady-state approximation to the population of the excited bound complex, ⟨∂*c*_(FO)*_/∂*t*⟩ = 0, and write its concentration as

8 $$\left\langle c_{\left(\mathrm{FQ}\right)*}\right\rangle =\frac{k_{b}}{k_{d}+k_{i}}\left\langle c_{F*}\right\rangle \left\langle c_{Q}\right\rangle$$

A steady-state condition for the concentration of the excited complex (eq 8) implies a quasi-equilibrium wherein an apparent equilibrium constant can be defined as

9 $$K_{\left(\mathrm{FQ}\right)*}^{\mathrm{ss}}=\frac{k_{b}}{k_{d}+k_{i}}c^{⌀}=\frac{\left\langle c_{\left(\mathrm{FQ}\right)*}\right\rangle }{\left\langle c_{F*}\right\rangle \left\langle c_{Q}\right\rangle }c^{⌀}$$

with *c* ^⌀^ the standard concentration, which is introduced to render *K*_(FQ)*_^ss^ dimensionless.

We continue by applying a steady-state approximation also for the concentration of F*

10 $$\left\langle \frac{\partial c_{F*}}{\partial t}\right\rangle =k_{a}\left\langle c_{F}\right\rangle -\left(k_{f}+k_{\mathrm{nr}}\right)\left\langle c_{F*}\right\rangle -k_{b}\left\langle c_{F*}\right\rangle \left\langle c_{Q}\right\rangle +k_{d}\left\langle c_{\left(\mathrm{FQ}\right)*}\right\rangle =0$$

and utilize the expression of ⟨*c*_(FQ)*_⟩ described in eq 8 to obtain

11 $$\left\langle c_{F*}\right\rangle =\frac{k_{a}}{k_{f}+k_{\mathrm{nr}}+k_{q}\left\langle c_{Q}\right\rangle }\left\langle c_{F}\right\rangle$$

with *k*_*q*_ an effective second-order rate constant defined by

12 $$k_{q}=k_{b}\left(1-\frac{k_{d}}{k_{d}+k_{i}}\right)$$

As expected, in the special case where *k*_*i*_ ≫*k*_*d*_, the effective rate constant, *k*_*q*_, approaches the binding rate constant *k*_*b*_. Note that it is customary in the literature to combine the chemical reactions in eq 6 and eq 7 into one complex reaction

13 $$F^{*}+Q\overset{k_{q}}{\to }F+Q$$

with *k*_*q*_ a second-order rate constant for this complex reaction (eq 13). Nonetheless, applying a steady-state approximation to *c*_F*_ and considering eq 13 instead of eq 6 and eq 7, yields exactly the expression written in eq 11.

The fluorescence intensity in the presence of quencher, *I*__Q__, is then proportional to

14 $$I_{Q}\propto \frac{k_{f}k_{a}}{k_{f}+k_{\mathrm{nr}}+k_{q}\left\langle c_{Q}\right\rangle }\langle c_{F}\rangle_{Q}$$

where the subscript “_Q_” of the angular brackets is introduced to emphasize that the average pertains to a system with a quencher. In principle, this term is distinguished from that of a system without quencher, such as ⟨*c*_F_⟩ in eq 5. Nonetheless, because the quencher can interact only with F*, under equal conditions of radiation and equal (total) amount of fluorophore, the concentrations of unbound ground-state fluorophore in both systems are assumed equal, that is, ⟨*c*_F_⟩_Q_ = ⟨*c*_F_⟩. As a consequence, the ratio of fluorescence intensity without quencher to that with quencher gives the famous Stern–Volmer equation,^1^

15 $$\frac{I_{{}^{\circ}}}{I_{Q}}=\frac{k_{f}+k_{\mathrm{nr}}+k_{q}\left\langle c_{Q}\right\rangle }{k_{f}+k_{\mathrm{nr}}}=1+\frac{k_{q}}{k_{f}+k_{\mathrm{nr}}}\left\langle c_{Q}\right\rangle =1+K_{\mathrm{SV}}\left\langle c_{Q}\right\rangle$$

which is linear as a function of ⟨*c*_Q_⟩. This linear relation has been found valid for a substantial number of experimental systems.^2–6^ The term *K*_SV_ ≡ *k*_*q*_/(*k*_*f*_ + *k*_nr_) is known as the Stern–Volmer constant.

### Static Quenching

In static quenching,^7–9^ it is the fluorophore in the ground state (and not in the excited state as in dynamic quenching) that forms a complex with the quencher,

16 $$F+Q\overset{k_{c}}{\underset{k_{u}}{⇌}}\mathrm{FQ},K_{\mathrm{FQ}}=\frac{k_{c}}{k_{u}}c^{⌀}$$

It is assumed that the complex can absorb light, in general, with a different frequency than the unbound fluorophore

17 $$\mathrm{FQ}\overset{+h\nu^{\prime \prime }}{\underset{k_{e}}{\to }}{\left(\mathrm{FQ}\right)}^{*},\mathrm{rate}=\left\langle \partial c_{\left(\mathrm{FQ}\right)*}/\partial t\right\rangle =k_{e}\left\langle c_{\mathrm{FQ}}\right\rangle$$

however, it can not emit light. In due course, the complex relaxes then only by nonradiative pathways

18 $${\left(\mathrm{FQ}\right)}^{*}\overset{k_{\mathrm{rx}}}{\to }F+Q,\mathrm{rate}=-\left\langle \frac{\partial c_{\left(\mathrm{FO}\right)*}}{\partial t}\right\rangle =k_{\mathrm{rx}}\left\langle c_{\left(\mathrm{FQ}\right)*}\right\rangle$$

Hence, relative to a system in which the quencher is not present, there is a decrease in fluorescence intensity. As before, the chemical reactions described in eqs 1–3 take place in the system, and because the quencher does not interact with the excited fluorophore, the steady-state result relating *c*_F*_ to *c*_F_ expressed in eq 4 holds here as well.

We apply a steady-state condition for the concentration of the ground-state complex

19 $$\left\langle \frac{\partial c_{\mathrm{FQ}}}{\partial t}\right\rangle =k_{c}\left\langle c_{F}\right\rangle \left\langle c_{Q}\right\rangle -k_{u}\left\langle c_{\mathrm{FQ}}\right\rangle -k_{e}\left\langle c_{\mathrm{FQ}}\right\rangle =0$$

and obtain

20 $$\left\langle c_{\mathrm{FQ}}\right\rangle =\frac{k_{c}\left\langle c_{F}\right\rangle \left\langle c_{Q}\right\rangle }{k_{u}+k_{e}}$$

which implies a steady-state association constant

21 $$K_{\mathrm{FQ}}^{\mathrm{ss}}=\frac{k_{c}}{k_{u}+k_{e}}c^{⌀}=\frac{\left\langle c_{\mathrm{FQ}}\right\rangle }{\left\langle c_{F}\right\rangle \left\langle c_{Q}\right\rangle }c^{⌀}$$

Given equal total fluorophore’s concentrations in systems with quencher (subscripted with “_*Q*_”), and without quencher (here subscripted with “_o_”), we can write

22 $$\langle c_{F}\rangle_{{}^{\circ}}+\langle c_{F*}\rangle_{{}^{\circ}}=\langle c_{F}\rangle_{Q}+\langle c_{F*}\rangle_{Q}+\langle c_{\mathrm{FQ}}\rangle_{Q}+\langle c_{\left(\mathrm{FQ}\right)*}\rangle_{Q}$$

and assume that |⟨*c*_F*_ $\rangle_{{}^{\circ}}$ – (⟨*c*_F*_⟩_Q_ + ⟨*c*_(FQ)*_⟩_Q_)|≪⟨*c*_F_ $\rangle_{{}^{\circ}}$ , which is valid for weak fluorescence absorptions, low quencher concentrations, or small values of *K*_FQ_. Consequently, the ratio between fluorescence intensity without quencher and that with quencher is

23 $$\frac{I_{{}^{\circ}}}{I_{Q}}=\frac{k_{f}\langle c_{F*}\rangle_{{}^{\circ}}}{k_{f}\langle c_{F*}\rangle_{Q}}=\frac{\frac{k_{a}}{k_{f}+k_{\mathrm{nr}}}\langle c_{F}\rangle_{{}^{\circ}}}{\frac{k_{a}}{k_{f}+k_{\mathrm{nr}}}\langle c_{F}\rangle_{Q}}=\frac{\langle c_{F}\rangle_{Q}+\langle c_{\mathrm{FQ}}\rangle_{Q}}{\langle c_{F}\rangle_{Q}}=1+\frac{k_{c}}{k_{u}+k_{e}}\left\langle c_{Q}\right\rangle =1+\frac{K_{\mathrm{FQ}}^{\mathrm{ss}}}{c^{⌀}}\left\langle c_{Q}\right\rangle$$

where we utilized the relation in eq 20. It is interesting to note that eq 23 has the same form as the Stern–Volmer relation for dynamic quenching expressed in eq 15, with *K*_SV_ substituted by *K*_FQ_^ss^/*c* ^⌀^. In the event that the rate of light absorption by the ground-state complex is negligible compared to the rate of complex unbinding, *k*_*e*_ ≪ *k*_*u*_ , *K*_FQ_^ss^ approaches *K*_FQ_. Many experimental reports confirm that also for static quenching, the plot of the ratio of fluorescence intensities, $I_{{}^{\circ}}$ /*I*_Q_, as a function of quencher’s concentration (customary referred to as SV plot) is linear.^10–13^ As is the case with dynamic quenching (eq 15), the quencher’s concentration appearing on the right-hand side of eq 23 is of its unbound state at ’equilibrium’. In the majority of studies, this concentration is taken as the total concentration of quencher introduced to the system, *c*_Q_^total^, which is justified for *c*_Q_^total^ ≫*c*_F_^total^. Several authors^14–16^ discussed this point for the case of static quenching and provided an alternative equation relating $I_{{}^{\circ}}$ /*I*_Q_ to *c*_Q_^total^ for all concentrations.

### Deviations of SV Plots from Linearity

As mentioned above, SV plots have been validated in many cases to be linear for both dynamic and static mechanisms.^17^ That said, there are as well, significant number of reports indicating deviations from this linear behavior.^18^ These deviations can be negative (i.e., with a downward curvature) or positive (upward curvature) relative to a straight line.^19^ Negative deviations of SV plots are normally attributed to (1) formation of an excited complex, (FQ)*, that can also emit light,^20^ (2) clustering of quencher’s molecules,^21,22^ and (3) saturation of fluorophore-quencher bound population.^23^

Several models were proposed to address positive deviations of SV plots.^24^ One explanation is the simultaneous action of dynamic and static mechanisms.^25^ In this case, it is assumed that the total reduction in fluorescence intensity reflected in the ratio $I_{{}^{\circ}}$ /*I*_Q_ (hereafter, we drop the subscript “Q” for the system with quencher), is expressed by the product of the corresponding reductions due to each of the individual quenching mechanisms,

24 $$\frac{I_{{}^{\circ}}}{I}=\left(1+K_{\mathrm{FQ}}^{\mathrm{ss}}\left\langle c_{Q}\right\rangle /c^{⌀}\right)\left(1+K_{\mathrm{SV}}\left\langle c_{Q}\right\rangle \right)$$

yielding a quadratic dependence of $I_{{}^{\circ}}$ /*I* on quencher’s concentration. Several reports in the literature fitted successfully observed upward curvatures of SV plots^3,26–30^ with eq 24, whereas others obtained equilibrium constants that are negative, and therefore, could not provide meaningful interpretation to the fitted parameters.^31–33^ Another model for upward deviations is due to Frank and Wawilow,^34^ who assumed that in addition to interactions operating within the collision sphere (defined by the fluorophore and quencher molecular sizes), an excited fluorophore can also interact with a quencher if both are present inside a sphere characterized by a larger volume, ω. This concept defines an additional route for quenching with a probability of 1 inside this “sphere of action”, and 0 otherwise. The Stern–Volmer equation is then said to be multiplied by an exponential factor to account for the probability of a quencher residing within volume ω around the excited fluorophore,

25 $$\frac{I_{{}^{\circ}}}{I}=\left(1+K_{\mathrm{SV}}\left\langle c_{Q}\right\rangle \right)\exp^{\omega N_{A}\left\langle c_{Q}\right\rangle }$$

where *N*_A_ is Avogadro’s number. The dependency of $I_{{}^{\circ}}$ /*I* on quencher’s concentration described in eq 25 has been found to fit well many experimental SV plots exhibiting positive deviations.^35–39^ Still, few reports raised some criticisms over this “sphere of action” mechanism, questioning its concept^40–42^ or the large values of the sphere’s radius inferred from the model.^17,43–45^ Other models addressing positive deviations from SV plots assume that one or more of the rate constants mentioned above depend on the age of the excited molecule^46^ or on reactants’ concentrations.^47–50^

In this paper, we derive an equation to quantify SV plots by assuming that each fluorophore is in a steady-state (quasi-equilibrium) with only a small set of quencher molecules located in its immediate vicinity. As a consequence, the expressions of bimolecular association rates need to include correlations in reactant concentrations. When this is applied, the resulting equations are capable of accounting for upward and downward curvatures in pure dynamic and pure static mechanisms. Partitioning the system into small subsystems naturally represents fluorophore-quencher interactions physically confined to restricted volumes. Hence, we examined this model on many experimental fluorescence quenching plots under various types of confinement and obtained excellent agreement. Interestingly, we found that this model accounts successfully also for deviations from linearity observed in homogeneous solutions. This is plausibly due to limited diffusion, triggering repetitive binding of the fluorophore with the same set of surrounding quenchers.

## Results and Discussion

### Dynamic Quenching of Single-Fluorophore Systems

In writing the expression of reaction rate for binding the excited fluorophore with quencher (eq 6), as well as the expression of the corresponding steady-state equilibrium constant (eq 9), we followed an assumption made in all works in the literature where reactants’ concentrations are decoupled. As a result, the uncorrelated product of each average, ⟨*c*_F*_⟩⟨*c*_Q_⟩, is considered. This assumption is valid in the thermodynamic limit, in which case, the numbers of fluorophore and quencher molecules are large, and all fluorophores in the system are able to interact with all quenchers. However, because fluorophore-quencher interactions are two-body in nature, in small systems (or in large systems characterized by many fluorophore-quencher subsystems, each confined to a small domain containing small numbers of particles), the concentrations of F* and Q are correlated.^51–55^ Therefore, the expression of the reaction rate ought to include the average of their product, ⟨*c*_F*_ ·*c*_Q_⟩, that is, eq 6 should read,

26 $$F^{*}+Q\overset{k_{b}}{\underset{k_{d}}{⇌}}{\left(\mathrm{FQ}\right)}^{*},\mathrm{rate}=-\left\langle \frac{\partial c_{F*}}{\partial t}\right\rangle =\left\langle \frac{\partial c_{\left(\mathrm{FO}\right)*}}{\partial t}\right\rangle =k_{b}\left\langle c_{F*}\cdot c_{Q}\right\rangle -k_{d}\left\langle c_{\left(\mathrm{FQ}\right)*}\right\rangle$$

By applying a steady-state condition to the concentration of the excited fluorophore,

27 $$\left\langle \frac{\partial c_{F*}}{\partial t}\right\rangle =k_{a}\left\langle c_{F}\right\rangle -\left(k_{f}+k_{\mathrm{nr}}\right)\left\langle c_{F*}\right\rangle -k_{b}\left\langle c_{F*}\cdot c_{Q}\right\rangle +k_{d}\left\langle c_{\left(\mathrm{FQ}\right)*}\right\rangle =0$$

we get,

28 $$\left\langle c_{F*}\right\rangle =\frac{k_{a}\left\langle c_{F}\right\rangle -k_{b}\left\langle c_{F*}\cdot c_{Q}\right\rangle +k_{d}\left\langle c_{\left(\mathrm{FQ}\right)*}\right\rangle }{k_{f}+k_{\mathrm{nr}}}$$

and a corresponding condition to the concentration of the excited complex yields

29 $$K_{\left(\mathrm{FQ}\right)*}^{\mathrm{ss}}=\frac{k_{b}}{k_{d}+k_{i}}c^{⌀}=\frac{\left\langle c_{\left(\mathrm{FQ}\right)*}\right\rangle }{\left\langle c_{F*}\cdot c_{Q}\right\rangle }c^{⌀}$$

instead of the steady-state constant expressed in eq 9. The fluorescence intensity in the presence of quencher is then

30 $$I\propto k_{f}\langle c_{F*}\rangle_{Q}=\frac{k_{f}k_{a}\langle c_{F}\rangle_{Q}}{k_{f}+k_{\mathrm{nr}}}\left[1+\frac{k_{d}\left\langle c_{\left(\mathrm{FQ}\right)*}\right\rangle -k_{b}\left\langle c_{F*}\cdot c_{Q}\right\rangle }{k_{a}\langle c_{F}\rangle_{Q}}\right]=\frac{k_{f}k_{a}\langle c_{F}\rangle_{Q}}{k_{f}+k_{\mathrm{nr}}}\left[1-\frac{k_{i}\left\langle c_{\left(\mathrm{FQ}\right)*}\right\rangle }{k_{a}\langle c_{F}\rangle_{Q}}\right]$$

where the last equality uses the relation in eq 29. As before, if fluorophore-quencher interactions are significant only when the fluorophore is electronically excited (thus, only the dynamic mechanism is operational), then to a very good approximation, ⟨*c*_F_⟩__Q__ = ⟨*c*_F_⟩, and hence, the ratio of fluorescence intensities, in the absence (eq 5) and presence (eq 30) of quencher is

31 $$\frac{I_{{}^{\circ}}}{I}={\left[1-\frac{k_{i}\left\langle c_{\left(\mathrm{FQ}\right)*}\right\rangle }{k_{a}\left\langle c_{F}\right\rangle }\right]}^{-1}$$

As expected, the ratio in eq 31 describes a reduction in fluorescence intensity upon the addition of quencher, because values of rate constants and concentrations are positive. However, the problem is to evaluate the average concentration of the excited bound complex, ⟨*c*_(FQ)*_⟩, or alternatively, the average of the coupled unbound concentrations, ⟨*c*_F*_ ·*c*_Q_⟩, in the small system (or subsystems). This problem can be solved relatively easily for the case in which the small system includes only one fluorophore, *N*_F_^total^=1, but still, with an arbitrary number of quencher molecules, *N*_Q_^total^. In this event, it is possible to express the average concentration of the excited complex by the value of the steady-state constant,^52,55^

32 $$\langle c_{\left(\mathrm{FQ}\right)*}\rangle_{N_{F}^{\mathrm{total}}=1}=\frac{N_{Q}^{\mathrm{total}}K_{\left(\mathrm{FQ}\right)*}^{\mathrm{ss}}}{V\left[Vc^{⌀}+N_{Q}^{\mathrm{total}}K_{\left(\mathrm{FQ}\right)*}^{\mathrm{ss}}\right]}=\frac{c_{Q}^{\mathrm{total}}K_{\left(\mathrm{FQ}\right)*}^{\mathrm{ss}}}{V\left[c^{⌀}+c_{Q}^{\mathrm{total}}K_{\left(\mathrm{FQ}\right)*}^{\mathrm{ss}}\right]}$$

where *V* is the volume of the small system, effectively defined by the space (volume) in which the group of quenchers participating in the small subsystem occupies.

We continue by inserting the relation described in eq 32 into eq 31 to evaluate the ratio of fluorescence intensities

33 $$\frac{I_{{}^{\circ}}}{I}={\left[1-\frac{k_{i}\frac{c_{Q}^{\mathrm{total}}K_{\left(\mathrm{FQ}\right)*}^{\mathrm{ss}}}{V\left[c^{⌀}+c_{Q}^{\mathrm{total}}K_{\left(\mathrm{FQ}\right)*}^{\mathrm{ss}}\right]}}{k_{a}\left\langle c_{F}\right\rangle }\right]}^{-1}={\left[1-\frac{k_{i}\frac{1}{V}}{k_{a}\left\langle c_{F}\right\rangle }\cdot \frac{1}{1+c^{⌀}/\left[K_{\left(\mathrm{FQ}\right)*}^{\mathrm{ss}}\cdot c_{Q}^{\mathrm{total}}\right]}\right]}^{-1}$$

For a series of experiments in which only the value of *c*_Q_^total^ varies, *k*_*i*_ and *k*_a_ are expected to be constant, and as argued above, so is ⟨*c*_F_⟩. This means we can define a constant $Z_{d}$ ,

34 $$Z_{d}\equiv \frac{k_{i}\frac{1}{V}}{k_{a}\left\langle c_{F}\right\rangle }=\frac{k_{i}c_{F}^{\mathrm{total}}}{k_{a}\left\langle c_{F}\right\rangle }=\frac{k_{i}}{k_{a}\left\langle N_{F}\right\rangle }$$

where 0 < ⟨*N*_F_⟩ < 1 is the probability of finding (or the fraction of time) the fluorophore (is) in its unbound ground state. Rewriting eq 33

35 $$\frac{I_{{}^{\circ}}}{I}={\left[1-\frac{Z_{d}}{1+c^{⌀}/\left(K_{\left(\mathrm{FQ}\right)*}^{\mathrm{ss}}\cdot c_{Q}^{\mathrm{total}}\right)}\right]}^{-1}=1+\frac{Z_{d}K_{\left(\mathrm{FQ}\right)*}^{\mathrm{ss}}\cdot c_{Q}^{\mathrm{total}}}{c^{⌀}+\left(1-Z_{d}\right)K_{\left(\mathrm{FQ}\right)*}^{\mathrm{ss}}\cdot c_{Q}^{\mathrm{total}}}$$

eq 35 describes a relation between $I_{{}^{\circ}}$ /*I* and *c*_Q_^total^, where the two terms, *K*_(FQ)*_^ss^ and $Z_{d}$ , can be considered as two parameters in a fitting procedure. Unlike the linear Stern–Volmer equation (eq 15), the dependency in eq 35 of $I_{{}^{\circ}}$ /*I* is on the total concentration of quencher, and its validity is not contingent on it being in excess relative to the fluorophore. In addition, the fitting gives directly the steady-state constant for binding.

### Static Quenching of Single-Fluorophore Systems

In deriving the equation for static quenching in the Introduction, we assumed the binding rate is proportional to decoupled fluorophore and quencher concentrations (eq 19), which was later propagated to the expression of *K*_FQ_^ss^ (eq 21). Again, this is valid only for large systems, and in order to describe association reactions in a small system (or in a large system composed of many noninteracting small subsystems), cross-correlations between reactants’ concentrations should be accounted for. That means, eq 21 should read,

36 $$K_{\mathrm{FQ}}^{\mathrm{ss}}=\frac{k_{c}}{k_{u}+k_{e}}c^{⌀}=\frac{\left\langle c_{\mathrm{FQ}}\right\rangle }{\left\langle c_{F}\cdot c_{Q}\right\rangle }c^{⌀}$$

Given the model of static quenching presented in the introduction and the assumptions therein, we continue from eq 23, and express the ratio of fluorescence intensities in the absence and presence of quencher as,

37 $$\frac{I_{{}^{\circ}}}{I}=\frac{k_{f}\langle c_{F*}\rangle_{{}^{\circ}}}{k_{f}\langle c_{F*}\rangle_{Q}}=\frac{\langle c_{F}\rangle_{{}^{\circ}}}{\langle c_{F}\rangle_{Q}}=1+\frac{\langle c_{\mathrm{FQ}}\rangle_{Q}}{\langle c_{F}\rangle_{Q}}=1+\frac{\langle c_{\mathrm{FQ}}\rangle_{Q}}{c_{F}^{\mathrm{total}}-\langle c_{F*}\rangle_{Q}-\langle c_{\mathrm{FQ}}\rangle_{Q}}$$

where in the denominator of the last term, we ignored the contribution of ⟨*c*_(FQ)*_⟩_Q_ to the total fluorophore’s concentration. As in the previous subsection for dynamic quenching, also here we assume a system with only one fluorophore (*N*_F_^total^=1), an arbitrary number of quencher molecules (*N*_Q_^total^), and relate the ground-state bound-complex concentration, ⟨*c*_FQ_⟩, to *K*_FQ_^ss^, *c*_Q_^total^, and *V* by,^52,55^

38 $$\langle c_{\left(\mathrm{FQ}\right)}\rangle_{N_{F}^{\mathrm{total}}=1}=\frac{c_{Q}^{\mathrm{total}}K_{\mathrm{FQ}}^{\mathrm{ss}}}{V\left[c^{⌀}+c_{Q}^{\mathrm{total}}K_{\mathrm{FQ}}^{\mathrm{ss}}\right]}$$

analogous to eq 32. To advance the derivation, we approximate ⟨*c*_F*_⟩_Q_ in terms of *c*_F_^total^ and ⟨*c*_FQ_⟩_Q_. A general and simple representation by these two concentrations is a linear combination,

39 $$\langle c_{F*}\rangle_{Q}≃\alpha c_{F}^{\mathrm{total}}-\beta \langle c_{\mathrm{FQ}}\rangle_{Q}$$

with 0 < α < 1 and β ≥ 0. Inserting eq 38 and the assumption made in eq 39 into eq 37 yields

40 $$\begin{array}{l} \frac{I_{{}^{\circ}}}{I}=1+\frac{\langle c_{\mathrm{FQ}}\rangle_{Q}}{\left(1-\alpha \right)c_{F}^{\mathrm{total}}+\left(\beta -1\right)\langle c_{\mathrm{FQ}}\rangle_{Q}}=1+\frac{K_{\mathrm{FQ}}^{\mathrm{ss}}\cdot c_{Q}^{\mathrm{total}}}{(1-\alpha )\left(c^{⌀}+K_{\mathrm{FQ}}^{\mathrm{ss}}\cdot c_{Q}^{\mathrm{total}}\right)+\left(\beta -1\right)K_{\mathrm{FQ}}^{\mathrm{ss}}\cdot c_{Q}^{\mathrm{total}}}\\ =1+\frac{\frac{1}{1-\alpha }K_{\mathrm{FQ}}^{\mathrm{ss}}\cdot c_{Q}^{\mathrm{total}}}{c^{⌀}+\frac{\beta -\alpha }{1-\alpha }K_{\mathrm{FQ}}^{\mathrm{ss}}\cdot c_{Q}^{\mathrm{total}}}=1+\frac{Z_{s}K_{\mathrm{FQ}}^{\mathrm{ss}}\cdot c_{Q}^{\mathrm{total}}}{c^{⌀}+{\mathrm{WZ}}_{s}K_{\mathrm{FQ}}^{\mathrm{ss}}\cdot c_{Q}^{\mathrm{total}}} \end{array}$$

where

41 $$Z_{s}\equiv \frac{1}{1-\alpha }$$

and W≡β−α>−1 . For β = α (i.e., W=0 ), the ratio of fluorescence intensities is a linear function of *c*_Q_^total^, similar to the SV-relation stated in eq 23, but with a slope of $Z_{s}K_{\mathrm{FQ}}^{\mathrm{ss}}/c^{⌀}$ instead of *K*_FQ_^ss^/*c* ^⌀^. Note, eq 40 can fit experimental SV plots by two parameters $Z_{s}K_{\mathrm{FQ}}$ (the product of two terms that are not independent) and W .

Aiming to further simplify the relation in eq 39, we analyzed SV plots operating by static quenching and found that in many cases of upward curvatures, β can be approximated as 0. Thus,

42 $$\langle c_{F*}\rangle_{Q}≃\alpha c_{F}^{\mathrm{total}}$$

which yields,

43 $$\frac{I_{{}^{\circ}}}{I}=1+\frac{Z_{s}K_{\mathrm{FQ}}^{\mathrm{ss}}\cdot c_{Q}^{\mathrm{total}}}{c^{⌀}+\left(1-Z_{s}\right)K_{\mathrm{FQ}}^{\mathrm{ss}}\cdot c_{Q}^{\mathrm{total}}}$$

where $Z_{s}$ is defined in eq 41. The expression in eq 43 can model experimental data points with two fitting parameters, *K*_FQ_^ss^ and $Z_{s}$ . By considering the latter as constant, we assume that with changes of quencher’s concentration, the induced changes in the value of α = ⟨*c*_F*_⟩_Q_/*c*_F_^total^, relative to the value of 1, can be ignored.

### A Unified Equation of Single-Fluorophore Quenching

It is interesting that despite applying different physical pictures and assumptions to derive dynamic and static (assuming eq 42) quenching equations for systems composed of many independent single-fluorophore subsystems (eq 35 and eq 43), the dependency of $I_{{}^{\circ}}$ /*I* on *c*_Q_^total^ is the same. We can then write a unified equation,

44 $$\frac{I_{{}^{\circ}}}{I}=1+\frac{ZKc_{Q}^{\mathrm{total}}}{c^{⌀}+\left(1-Z\right)Kc_{Q}^{\mathrm{total}}}$$

describing the reduction in fluorescence intensity upon quenching, with $Z=Z_{d}$ (eq 34) and *K* = *K*_(FQ)*_^ss^ for dynamic mechanism, and with $Z=Z_{s}$ (eq 41) and *K* = *K*_FQ_^ss^ for static mechanism.

The relation between fluorescence intensities and quencher concentration described in eq 44 is, in general, nonlinear. Upward curvatures are observed for Z>1 (Figure 1a), whereas downward curvatures correspond to Z<1 (Figure 1b). Yet, $I_{{}^{\circ}}$ /*I* is linear as a function of *c*_Q_^total^ for cases in which the term $\left(1-Z\right){\mathrm{Kc}}_{Q}^{\mathrm{total}}$ is much smaller than *c* ^⌀^. This obviously holds when Z approaches 1 (Figure 1c), or when *K*, and/or *c*_Q_^total^, are small enough (Figure 1d). The latter two conditions are known to diminish correlations (couplings) between concentrations of reactants in binding reactions at finite systems.^52,53,55^ As a result, the behavior of the system approaches that of a macroscopic system, and thereby, eq 44 reduces to the well-known Stern–Volmer equation (eq 15 or eq 23). We would like to point out that even if the overall behavior of eq 44 is nonlinear, at sufficiently low quencher concentrations, the dependency is apparently linear (see blue and green lines in Figure 1d, relative to the corresponding curves in Figure 1a,1b), an observation frequently encountered in experimental SV plots.

**Figure 1:**
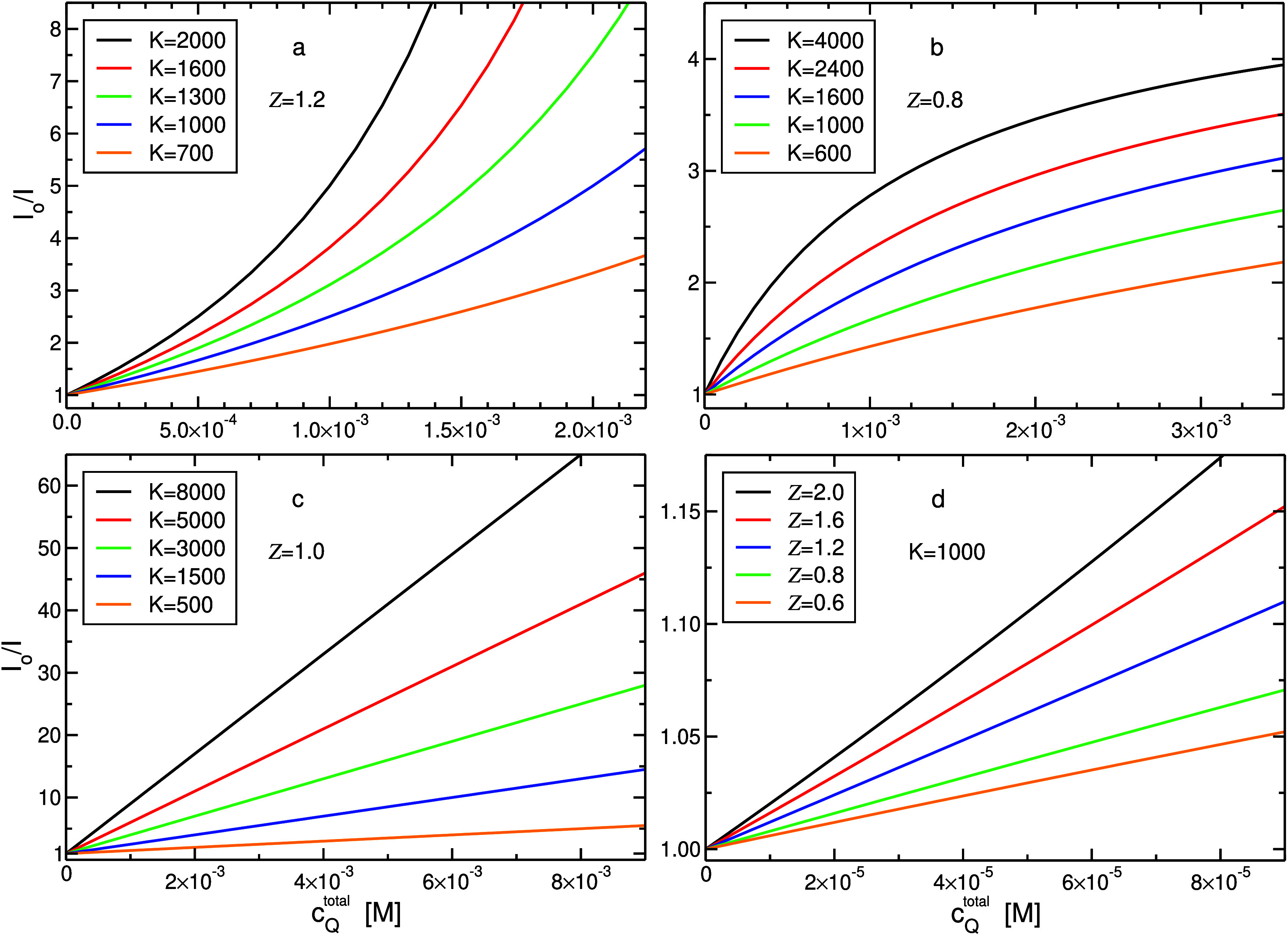
The ratio $I_{{}^{\circ}}/I$ calculated by eq 44 (*c* ^⌀^= 1 *M*), as a function of total quencher concentration, with (a) Z=1.2 , (b) Z=0.8, and (c) Z=1.0 , for several values of *K*, as well as, for (d) *K* = 1000 for several values of Z .

What is the physical meaning of a system characterized by Z larger, equal, or smaller than 1? For dynamic quenching, the numerator of $Z_{d}$ (eq 34), *k*_*i*_ *c*_F_^total^, expresses the largest possible rate to relax nonradiatively the excited complex (eq 7), when the quencher is added in excess relative to the fluorophore. In other words, it is the rate of relaxation-dissociation of the excited complex assuming its average concentration equals *c*_F_^total^. The term in the denominator is the rate of exciting the ground-state unbound fluorophore. Hence, Z larger, equal, or smaller than 1 means the maximal rate of quenching is larger, equal, or smaller, respectively, than the rate of excitation.

Note that because quenching requires the ratio of fluorescence intensities to be larger than (or equal to) 1, there is a condition between the values of *c*_Q_^total^, *K*, and Z , that should be satisfied. That is, $\left(Z-1\right){\mathrm{Kc}}_{Q}^{\mathrm{total}}<c^{⌀}$ , which is relevant only when Z>1 , thus, for upward curvatures. For downward curvatures in dynamic quenching, $Z_{d}<1$ (but still positive as required by its definition), so the condition $I_{{}^{\circ}}$ /*I* ≥ 1 is always satisfied. In static quenching, $Z_{s}$ should be larger than 1 (because α, defined in eq 42, can not be negative), and as a consequence, the assumption made in eq 42 can not support downward curvatures. Therefore, to model downward curvatures driven by a static mechanism, the assumption in eq 39 should be considered, and consequently, the relation in eq 40 ought to be applied. Modeling SV plots exhibiting downward ( W>0 ) and upward ( W<0 ) curvatures, as well as a straight line ( W=0 ), by eq 40 are shown in Figure 2.

**Figure 2:**
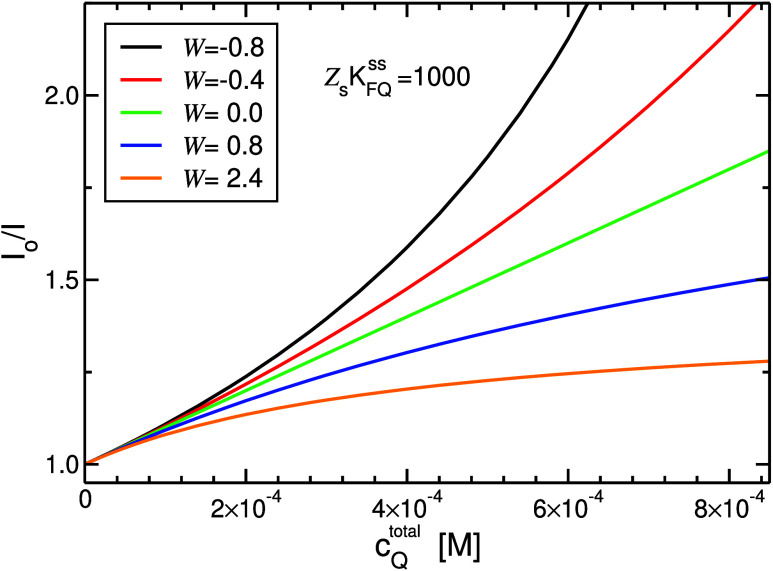
$I_{{}^{\circ}}$ /*I* as a function of *c*_Q_^total^ for static quenching according to eq 40, where it is assumed ⟨*c*_F*_⟩_Q_ = *α c*_F_^total^ – β ⟨*c*_FQ_⟩_Q_. Downward curvatures correspond to positive, whereas upward curvatures to negative, values of W≡β−α .

### Examining the Single-Fluorophore Quenching Model on Experimental SV Plots

To test the theory, we fit the proposed equations to experimental fluorescence quenching data reported in the literature. All fittings were performed by Grace plotting software, version 5.1.25. Except of few cases in which the data points were published in a table, or were provided by the authors, we extracted the values of the points from the published figures by the Engauge Digitizer software.^56^ If not included in the data set, the point [0,1] was introduced by definition. In all figures, symbols are experimental data points and solid lines are fittings of eq 44. This does not include SV plots displaying downward curvatures by static quenching, in which case, the solid lines are fittings of eq 40. When a macroscopic system forms an ensemble of spatially restricted subsystems, for example, a micellar solution with the micelles confining the fluorophores and quenchers, the local concentration of the quencher is, in general, not known. Some authors performed an analysis to estimate the local effective concentration, whereas others did not. In any case, we always considered only the values presented in the reported SV plots. Note that because in eq 44 the steady-state constant, *K*, and total quencher’s concentration of the subsystem, *c*_Q_^total^, always appear as a product, scaling *c*_Q_^total^ by a factor will merely modify *K* by the inverse of that factor, whereas Z will remain unchanged.

We start by applying eq 44 to fluorescence quenched by a dynamic mechanism^57–63^ (SI section SI-1). It is straightforward to attribute quenching to a pure dynamic mechanism when the time-resolved measurements, $\tau_{{}^{\circ}}$ /τ vs *c*_Q_^total^, display the same linear relation as that obtained by steady-state measurements. Although we also included these cases in our analyses,^57–59,62^ this condition restricts the data points to a straight line, which is already described well by the SV equation. In many reports, additional involvement of a static mechanism is concluded solely on the basis that the SV plot displays positive deviations from linearity. Because in this paper we argue that upward and downward curvatures are possible for purely dynamic (or purely static) mechanism, we ignore such interpretations, unless evidence of involvement of a static mechanism is provided, such as a noticeable quencher-induced change in absorption spectra or a decrease of *K* with an increase in temperature. The SV plots operated by the dynamic mechanism we consider (Figures S1.1 and S1.2) exhibit linear relations, as well as, upward and downward curvatures, and the results of the fittings are very good (Table S1.1).

We continue the model’s evaluation on SV plots governed by static quenching^32,42,64–69^ (SI-2). In all cases, a static mechanism was concluded by the authors because the fluorescence’s lifetime with quencher (τ) was equal to that in the absence of quencher ( $\tau_{{}^{\circ}}$ ), except for one study where time-resolved measurements were not performed, and the conclusion was based on demonstrating the formation of a ground-state complex.^69^ To fit SV plots with upward curvatures, we utilized the unified equation, eq 44 (Figures S2.1, S2.2a, and Table S2.1). In contrast, SV plots with downward curvatures were fitted by eq 40 (Figure S2.2b–d and Table S2.2). It is worth commenting few points. In the work of Shaw et al.^42^ (Figure S2.1b), the Benesi–Hildebrand method was applied by the authors to determine *K*_FQ_^ss^ = 28.47. This value is in reasonable agreement with *K*_FQ_^ss^ = 18.5 we obtained from the fitting of the SV plot. Furthermore, in the work of Hollett et al.^32^ (Figure S2.1c), quenching of fluorescence was recorded at three different emission wavelengths. Upon fitting, we obtained *K*_FQ_^ss^ values that are aligned with the efficiency of the quenching displayed in the SV plots. Different emission wavelengths are attributed to different nanocrystallite sizes in the material; however, it is not clear whether these different sizes of the photoluminescent centers are characterized by different binding constants. In case they do not, we also calculated the geometric average of *K*_FQ_^ss^ derived from these three different wavelengths, and used this value as a constant for a one-parameter fitting (dashed lines in Figure S2.1c and gray entries in Table S2.1). As judged by the correlation coefficient, the obtained fitting is slightly less good for the 850 nm wavelength but still very reasonable. When fitting downward curvatures by static quenching (eq 40), it is the product $Z_{s}K_{\mathrm{FQ}}^{\mathrm{ss}}$ , and not *K*_FQ_^ss^ on its own, that is extracted from the fitting. In their study, Ranjit and Levitus^67^ employed three different nucleotide phosphates to quench the dye’s fluorescence. The value of $Z_{s}K_{\mathrm{FQ}}^{\mathrm{ss}}$ obtained from the fittings increases with the efficiency of quenching displayed in the SV plots, whereas the value of W does not vary substantially.

Next, we examine SV plots in which the quenching is likely a combination of both dynamic and static mechanisms. In the models presented above for the pure mechanisms, the equations already include two fitting parameters. Obviously, in a model that describes simultaneous quenching by both mechanisms, the number of fitting parameters is expected to be larger. In section SI-12 we propose such a model. We start from a dynamic quenching in which the unbound ground-state fluorophore is subject to an additional equilibrium reaction with the quencher to form the ground-state complex (quantified by *K*_FQ_^ss^). The resulting equation, eq S4, is a generalization of dynamic quenching, that is, it reduces to eq 35 for *K*_FQ_^ss^→0. It contains four fitting parameters, and when attempted to be applied to experimental SV plots, the obtained results depended on the initial guess and on the fitting method. Therefore, instead of utilizing eq S4, we applied the unified equation (eq 44) to model quenching by combined mechanisms^28,29,70,71^ (SI-3). In this case, it is understood that the parameters obtained from the fittings are apparent (effective) quantities that, in the absence of a dominating mechanism, can not be directly interpreted. The results of the fittings (SI-3) are quite good, which might indicate that one mechanism is dominating the quenching behavior. We note that in the work of Bharadwaj et al.^70^ (Figure S3.1b), the three CdTe quantum dots are characterized by different sizes (d_QD3_ > d_QD2_ > d_QD1_) and the values of *K* obtained from the fittings follow the experimentally observed, nonmonotonic, quenching efficiency (whereas the value of Z is almost constant).

In the following, we continue the evaluation of the model (eq 44) on fluorophore-quencher systems under various confinements. That is, fluorophores embedded in micellar structures of Triton X-100 ^72–75^ and SDS^59,76–78^ (SI-4), lipid membranes^79–84^ (SI-5), conjugated polymers and block copolymers^43,85–91^ (SI-6), metal organic frameworks^92–95^ (SI-7), β-cyclodextrin and amylose structures^96–100^ (SI-8), microemulsions^101–104^ (SI-9), and proteins^33,105–107^ (SI-10). Finally, we also apply eq 44 to fluorescence quenching in homogeneous, organic^108–110^ and aqueous,^48,111–113^ solutions that exhibited either upward or downward curvature (SI-11). In total, we analyzed 151 SV plots. The results of fitting are excellent with an average correlation coefficient of 0.9985 and standard deviation of 0.0020, which provides validation of the localized and independent fluorescence quenching model proposed to compartmentalized (confined) systems. It is interesting, however, that even when applying this model to SV plots conducted in homogeneous solutions, the results are also very good. We suggest that during the time periods between successive associations of the fluorophore with quenchers (including the nonradiative relaxation processes of the bound complex), the diffusive distance of the molecules is small enough so that the fluorophore binds the same set of nearby quenchers. This effectively partitions the macroscopic system into the small independent subsystems we referred to in our model.

As mentioned above, at low enough quencher’s concentrations, experimental SV plots often display a linear behavior which is reproduced mathematically in eq 44. In several studies, the authors analyzed this regime and calculated the value of *K*_SV_ considering only this low concentration linear range. For these cases (26 in total), we calculated the value of ZK , obtained from the fitting to the entire nonlinear SV plot, and compared it to the reported *K*_SV_ at low concentrations. The results are presented in Tables S13.1 and S13.2. In the vast majority of the studies, the agreement is very good. Yet, in two cases,^66,91^ allyloxy-based MOF quenched by Pd^2+^ and Ur-PMMA-*b*-PVAc block copolymer quenched by Fe^3+^, there is a substantial discrepancy of a factor of 20 and 10, respectively. One explanation is that in these two cases, the effective local concentration of quencher, at low and high concentrations, is not related to *c*_Q_^total^ by the same factor.

It is important to note that in time-resolved measurements, average concentrations are irrelevant, and thereby, couplings (correlations) in reactants’ concentrations do not affect the behavior. That means, $I_{{}^{\circ}}$ /*I* vs *c*_Q_^total^ can exhibit nonlinearity while $\tau_{{}^{\circ}}$ /τ vs *c*_Q_^total^ is linear.

### Systems with Exciplex Emissions

When presenting the dynamic mechanism in the Introduction and, later, in Results and Discussion sections, we assumed the excited complex (exciplex), (FQ)*, does not emit light. However, there are systems in which additional emission by exciplex does take place.^114–116^ In this subsection, we show that even with this extra emission by the exciplex, the resulting SV plots still obey eq 44.

We consider a system adhering to the chemical reactions and corresponding relations described in eq 1–5, eq 7, and eq 26–28, subject to an additional process of exciplex emission

45 $${\left(\mathrm{FQ}\right)}^{*}\overset{-h\nu^{\prime \prime \prime }}{\underset{k_{\mathrm{ee}}}{\to }}F+Q,\mathrm{rate}=-\left\langle \frac{\partial c_{\left(\mathrm{FO}\right)*}}{\partial t}\right\rangle =k_{\mathrm{ee}}\left\langle c_{\left(\mathrm{FQ}\right)*}\right\rangle$$

In this model, application of a steady-state condition to ⟨*c*_(FO)*_⟩ gives

46 $$k_{b}\left\langle c_{F*}\cdot c_{Q}\right\rangle -k_{d}\left\langle c_{\left(\mathrm{FQ}\right)*}\right\rangle -k_{i}\left\langle c_{\left(\mathrm{FQ}\right)*}\right\rangle -k_{\mathrm{ee}}\left\langle c_{\left(\mathrm{FQ}\right)*}\right\rangle =0$$

from which we can define a steady-state constant for (fluorescent) exciplex formation

47 $$K_{\left(\mathrm{FQ}\right)*}^{\mathrm{ss}}=\frac{k_{b}}{k_{d}+k_{i}+k_{\mathrm{ee}}}c^{⌀}=\frac{\left\langle c_{\left(\mathrm{FQ}\right)*}\right\rangle }{\left\langle c_{F*}\cdot c_{Q}\right\rangle }c^{⌀}$$

There are fluorophore-quencher systems in which it is possible to differentiate the fluorescence emitted exclusively by the exciplex, *I* ^*ee*^, from that emitted by the unbound excited fluorophore, *I* ^*f*^. In this case, it is easy to show that application of the single-fluorophore quenching model results in a relation between $I_{{}^{\circ}}/I^{f}$ and *c*_Q_^total^ given by eq 44, with *K* expressed by eq 47 and Z by eq 34. Yet, in other cases, the emission spectra of F* and (FQ)* substantially overlap, and hence, both contribute to the observed fluorescence intensity, *I* ^*f*+*ee*^, which is then expressed by,

48 $$I^{f+\mathrm{ee}}\propto k_{f}\left\langle c_{F*}\right\rangle +k_{\mathrm{ee}}\left\langle c_{\left(\mathrm{FQ}\right)*}\right\rangle =\frac{k_{f}k_{a}\langle c_{F}\rangle_{Q}}{k_{f}+k_{\mathrm{nr}}}\left[1-\frac{k_{i}\left\langle c_{\left(\mathrm{FQ}\right)*}\right\rangle }{k_{a}\langle c_{F}\rangle_{Q}}\right]+k_{\mathrm{ee}}\left\langle c_{\left(\mathrm{FQ}\right)*}\right\rangle$$

where the contribution from the fluorescence of F* was taken from eq 30. Given the expression of $I_{{}^{\circ}}$ in eq 5 and assuming (as before for dynamic mechanisms) ⟨*c*_F_⟩_Q_ = ⟨*c*_F_⟩, the ratio of fluorescence intensities is,

49 $$\frac{I_{{}^{\circ}}}{I^{f+\mathrm{ee}}}={\left[1-\left(\frac{k_{i}}{k_{a}}+\frac{\left(k_{f}+k_{\mathrm{nr}}\right)k_{\mathrm{ee}}}{k_{f}k_{a}}\right)\frac{\left\langle c_{\left(\mathrm{FQ}\right)*}\right\rangle }{\left\langle c_{F}\right\rangle }\right]}^{-1}$$

The relation in eq 49 has the same form as that of eq 31. By applying the single-fluorophore quenching model, that is, expressing ⟨*c*_(FQ)*_⟩ by eq 32, it follows that the dependency of $I_{{}^{\circ}}/I^{f+ee}$ on *c*_Q_^total^ is also obeying eq 44, with *K* described in eq 47 and Z equals,

50 $$Z_{\mathrm{ee}}\equiv \left(\frac{k_{i}}{k_{a}}+\frac{\left(k_{f}+k_{\mathrm{nr}}\right)k_{\mathrm{ee}}}{k_{f}k_{a}}\right)\frac{c_{F}^{\mathrm{total}}}{\left\langle c_{F}\right\rangle }=\left(\frac{k_{i}}{k_{a}}+\frac{\left(k_{f}+k_{\mathrm{nr}}\right)k_{\mathrm{ee}}}{k_{f}k_{a}}\right)\frac{1}{\left\langle N_{F}\right\rangle }$$

## Conclusions

Deviations of fluorescence quenching data from the linear behavior predicted by the Stern–Volmer equation have been reported extensively in the literature. The accepted dogma is that linearity is observed only when a single mechanism drives the quenching process, whereas positive deviations are attributed for simultaneous action of dynamic and static mechanisms. Nevertheless, in many cases, this explanation is not supported by other, independent, criteria for a dual quenching scenario. In some reports, it is quite the opposite; involvement of a second mechanism is ruled-out. In this paper, we derive an equation to quantify the reduction in fluorescence intensity under steady-state conditions when a quencher is introduced into the system. We assume the same chemical reactions and processes as those leading to the Stern–Volmer equation for dynamic and static mechanisms. However, instead of assuming that all fluorophores are in quasi-equilibrium with all quenchers in the system, we hypothesize that each fluorophore is in quasi-equilibrium with only a small set of quenchers present in its close proximity. This divides the whole system into many small subsystems considered to be independent. The difference in considering many small subsystems as opposed to one large system is in the expressions of two-body interactions. Because there are only finite numbers of particles in each of the subsystems, the second-order reaction rates in eq 6 and eq 19 should be expressed by the average of the product of fluorophore and quencher concentrations, and not by (the common expression of) the product of the average of each concentration.^52–55^ As a consequence, the “equilibrium” concentration of fluorophore-quencher complex in a small system is different than that in a large system even if these two systems contain the same (total) concentrations of fluorophore and quencher molecules. By taking into account these correlations (couplings) between reactants’ concentrations, we obtained a relation, eq 44, that when using a (steady-state) fluorophore-quencher association constant, *K*, in units of *M* ^–1^, has the form,

51 $$\frac{I_{{}^{\circ}}}{I}=1+\frac{ZK{\left[Q\right]}_{T}}{1+\left(1-Z\right)K{\left[Q\right]}_{T}}$$

with [Q]__*T*__ the total concentration of quencher in the subsystem, and Z , a dimensionless quantity given in eq 34 and eq 41 for dynamic and static mechanisms, respectively. Depending on the system’s conditions, the ratio $I_{{}^{\circ}}$ /*I* as a function of [Q]__*T*__ can exhibit linear behavior, as well as upward ( Z>1 ) and downward ( Z<1 ) curvatures, even when only a single mechanism is operational. Still, downward curvatures induced by a static mechanism should be described by eq 40 instead, because in static quenching, $Z_{s}$ is physically meaningful only when it is equal to or larger than 1. We note that when the quencher concentration is low enough, or alternatively when Z∼1 , the relation reduces to the linear Stern–Volmer equation, with ZK equals *K*_SV_. From the expression of $Z_{d}$ in dynamic quenching, it is evident that upward (downward) curvatures of SV plots are seen when the maximum rate of quenching is larger (smaller) than the production rate of the excited fluorophore.

The proposed model, in which the system is partitioned into subsystems each containing a single fluorophore with nearby surrounding quenchers, represents a collection of spatially confined fluorescence quenching activities. We therefore utilized eq 44 (or eq 40 for downward curvatures by static mechanisms) as a two-parameter fitting equation to model experimental fluorescence quenching data (reported in the literature) of a large variety of systems in which the fluorophore, and likely the quencher, are under various types of translational restrictions. These include micelles, metal–organic frameworks, polymers, microemulsions, lipid membranes, and nanoparticles. The results of the fitting are very good. In cases in which the low-concentration regime exhibited a linear behavior, the value of *K*_SV_ extracted from the Stern–Volmer equation by the authors is similar to the value of ZK we obtained from the fitting of the entire concentration range. Intriguingly, we also applied this model to quenching data of fluorophores and quenchers that are dissolved in organic or aqueous, homogeneous solutions. The results were excellent as well. We conjecture that within time periods of binding cycles, quenchers are not able to diffuse far enough from their partner fluorophore, and a ’bounding’ force exists (albeit with a small magnitude) to keep the same group of quenchers around the fluorophore.

Last, remarkably, the quenching equation we propose (in either form, eq 51 or eq 44) can also describe reductions of fluorescence intensities in systems where the fluorophore-quencher excited complex is fluorescent.

## Supplementary Material



## Notes

Evaluation of the model on 151 experimental SV plots (SI-1–SI-11); derivation of simultaneous dynamic and static quenching of single-fluorophore systems (SI-12); comparison of the values of ZK obtained from fitting the model with experimental *K*_SV_ values extracted at low quencher’s concentrations (SI-13) (PDF)
