# Supplementary material for: Quenching Single-Fluorophore Systems and the Emergence of Nonlinear Stern–Volmer Plots
Source: ACS Omega. 2026 Feb 9;11(7):12713–24. doi: 10.1021/acsomega.5c13199 (PMC12947201; doi:10.1021/acsomega.5c13199)
Supplement: Supplementary file 1 [file ao5c13199_si_001.pdf]

Supporting Information: Quenching Single-Fluorophore Systems and the Emergence of Non-linear Stern-Volmer Plots

Ronen Zangi*[1,2,3]

[1] *Department of Organic Chemistry I, University of the Basque Country UPV/EHU, 20018 Donostia-San Sebastián, Spain*

[2] *Donostia International Physics Center (DIPC), 20018 Donostia-San Sebastián, Spain*

[3] *IKERBASQUE, Basque Foundation for Science, 48009 Bilbao, Spain*

December 16, 2025

*r.zangi@ikerbasque.org

SI-1 Testing Single-Fluorophore Quenching Model: Dynamic Mechanism

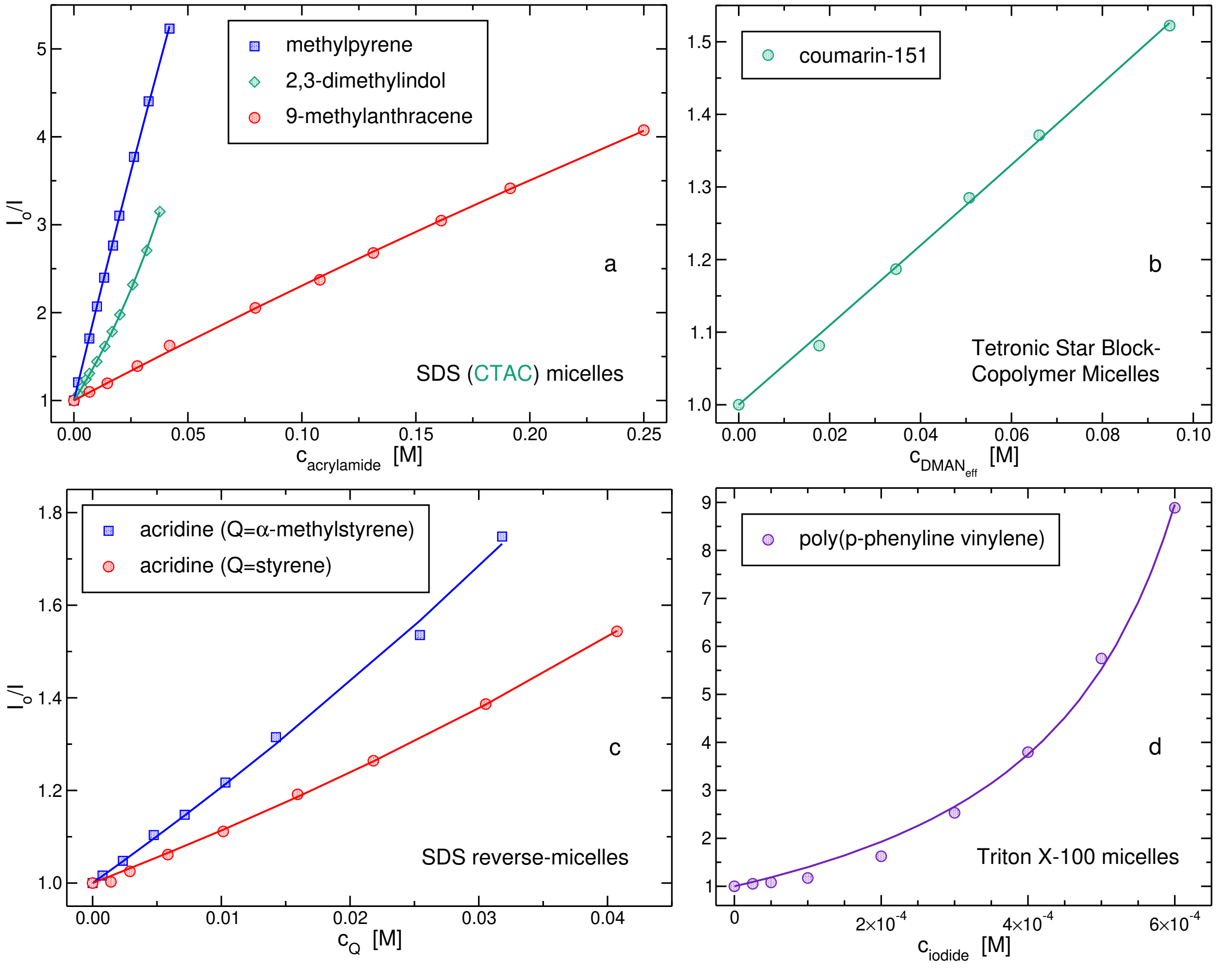

Figure S1.1: Fitting of Eq. 44 (Eq. 35) to fluorescence quenching plots governed by a dynamic mechanism where fluorophore and quencher are confined (for the duration of the measurements) to different types of micellar assemblies. The experimental data were taken from literature reports of (a) Rubio and Lissi[57], (b) Samanta et al.[58], (c) Buchviser and Gehlen[59], and (d) Sharma (where the points are listed in a table)[60]. Details of the fittings results are given in Table S1.1.

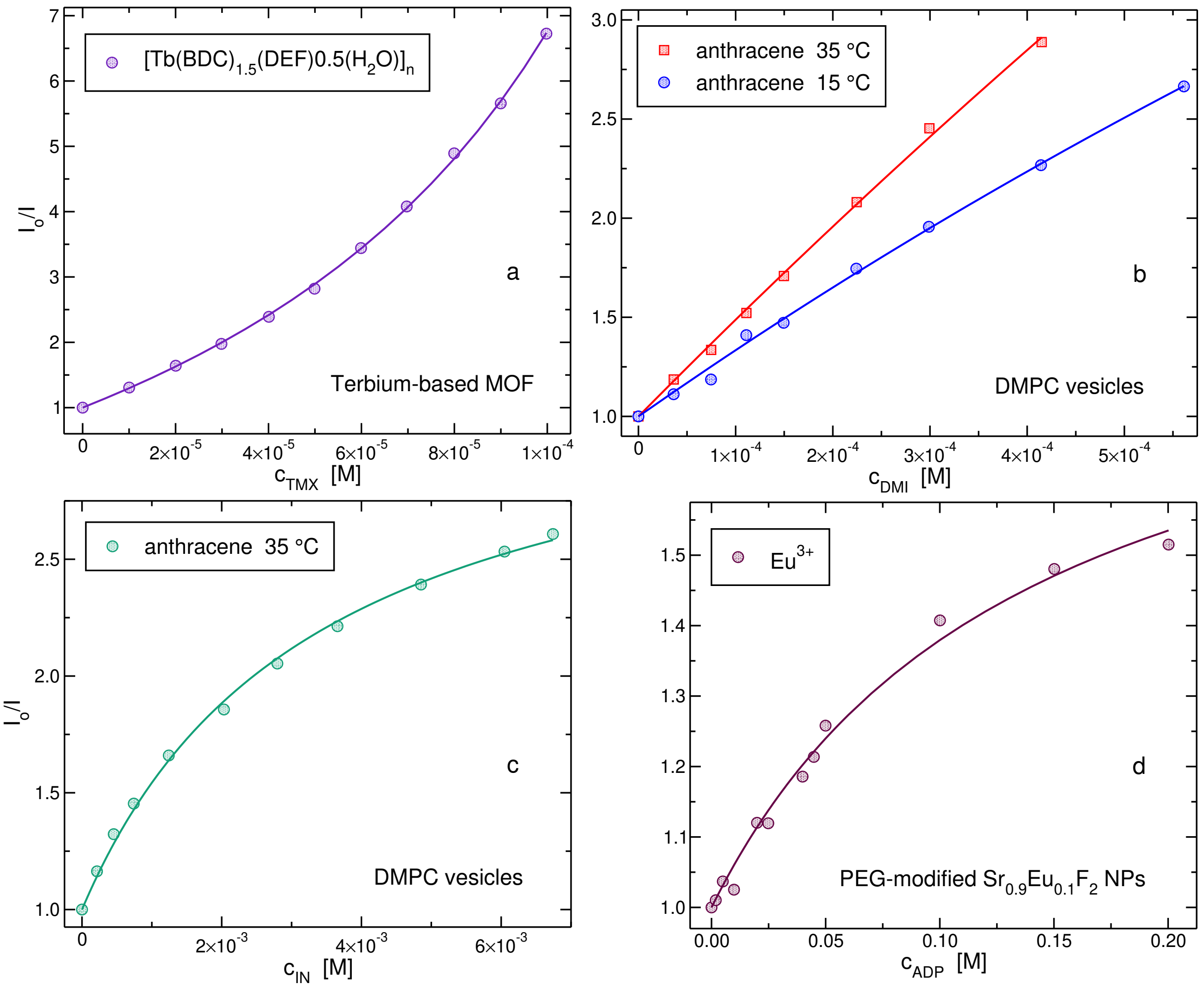

Figure S1.2: Fitting of Eq. 44 (Eq. 35) to SV plots governed by a dynamic mechanism. (a) Fluorescence of terbium-based metal-organic-framework (BDC = 1,4-benzene dicarboxylate, DEF = N,N-diethylformamide) quenched by thiamethoxam (Liu et al.[61]). Fluorescence of anthracene confined to DMPC vesicles (Novaira and Previtali[62]) quenched by (b) 1,2-dimethylindole and (c) indole. (d) Fluorescence of Eu^{3+}-doped SrF_2, PEG-modified, nanoparticles quenched by ammonium dihydrogen phosphate (Far et al.[63]). Details of the fittings results are provided in Table S1.1.

Table S1.1: Results of fitting Eq. 44 (Eq. 35) to fluorescence quenching data, shown in Fig. S1.1 and Fig S1.2, wherein the quenching mechanism is dynamic, and the fluorophore-quencher systems are subject to different types of confinement. R is the correlation coefficient of the fitting.

Fluorophore	Quencher	plotted in	$K^{ss}_{(FQ)^*}$	$\mathscr{Z}_d$	R
methylpyrene (SDS)[57]	acrylamide	Fig. S1.1a	110	0.985	0.9998
2,3-dimethylindol (CTAC)[57]	acrylamide	Fig. S1.1a	33.8	1.22	0.9999
9-methylanthracene (SDS)[57]	acrylamide	Fig. S1.1a	14.1	0.968	0.9998
C151 (copolymers)[58]	N,N-dimethylaniline	Fig. S1.1b	5.24	1.04	0.9992
acridine (SDS-reverse)[59]	α-methylstyrene	Fig. S1.1c	15.3	1.29	0.9986
acridine (SDS-reverse)[59]	styrene	Fig. S1.1c	6.12	1.77	0.9997
PPV (TX100)[60]	iodide	Fig. S1.1d	2270	1.54	0.9986
Terbium-based MOF[61]	thiamethoxam	Fig. S1.2a	$2.31 \cdot 10^4$	1.22	0.9998
ANT 35°C (DMPC vesicles)[62]	1,2-dimethylindole	Fig. S1.2b	5150	0.964	0.9993
ANT 15°C (DMPC vesicles)[62]	1,2-dimethylindole	Fig. S1.2b	3710	0.925	0.9987
ANT 35°C (DMPC vesicles)[62]	indole	Fig. S1.2c	1000	0.703	0.9992
Eu^{3+} (doped SrF_2 PEG-NP)[63]	ADP	Fig. S1.2d	13.7	0.475	0.9954

SI-2 Testing Single-Fluorophore Quenching Model: Static Mechanism

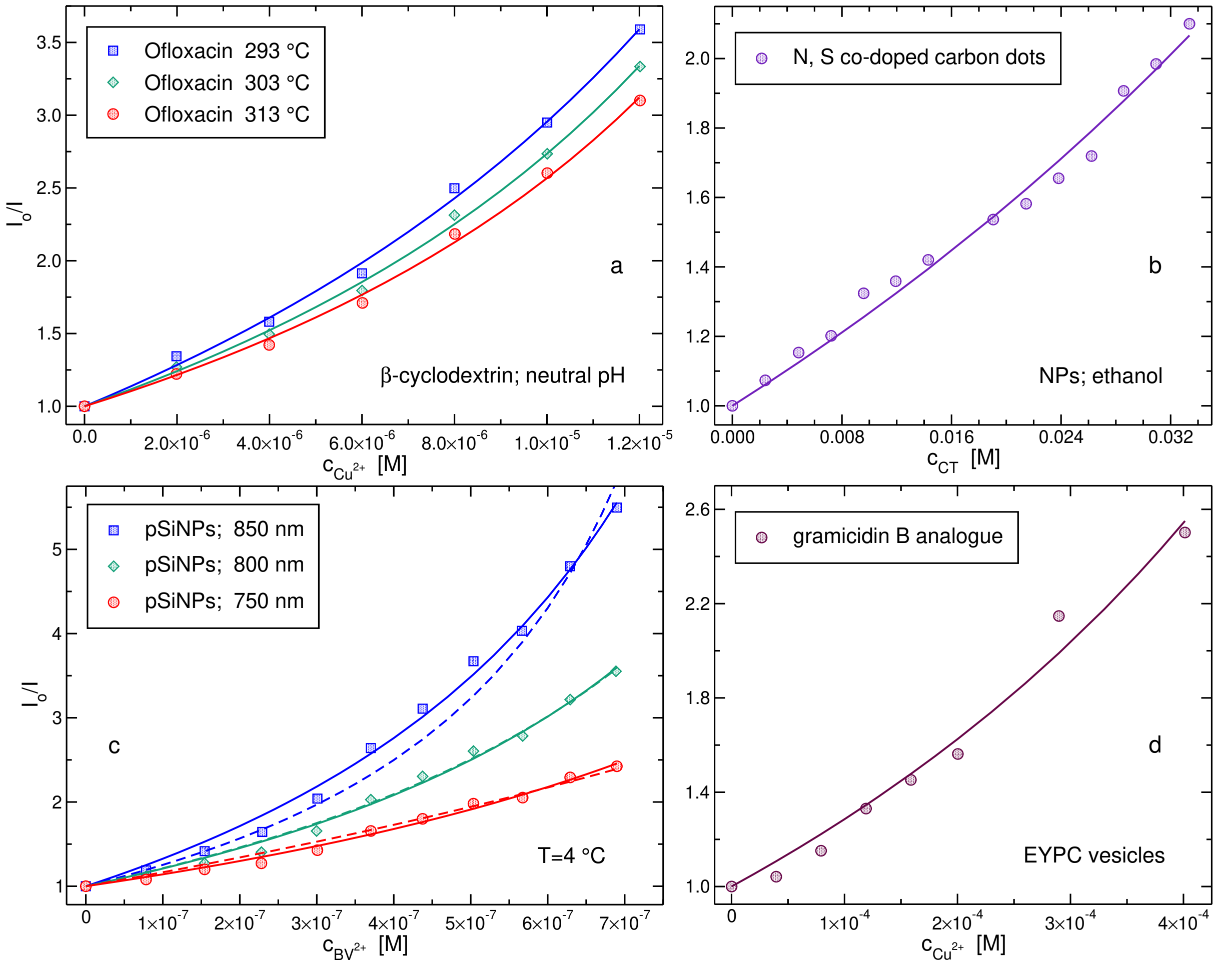

Figure S2.1: Fitting of Eq. 44 (Eq. 43) to fluorescence quenching plots governed by a static mechanism where fluorophore and quencher are under different types of confinements. (a) Ofloxacin, confined inside β-cyclodextrin under neutral pH, quenched by copper(II) at different temperatures (Padhan et al.[64]), (b) N, S co-doped carbon dots (nano-particles) in ethanol quenched by crotonaldehyde, CT (Shaw et al.[42]), (c) porous silicon nano-particles (pSiNPs) quenched by benzyl viologen (BV^{2+}) at three different emission wavelengths (Hollett et al.[32]), and (d) α-aminoisobutyric acid analogue of gramicidin B, in large unilamellar EYPC vesicles, quenched by copper (II) (Jelokhani-Niaraki et al.[65]). The dashed-lines in (c) are fitting results with a fixed value of K_{FQ}^{ss} for all emission wavelengths, where K_{FQ}^{ss} was determined by a geometric average of the values attained by the two-parameter fittings. Details of the fittings results are given in Table S2.1.

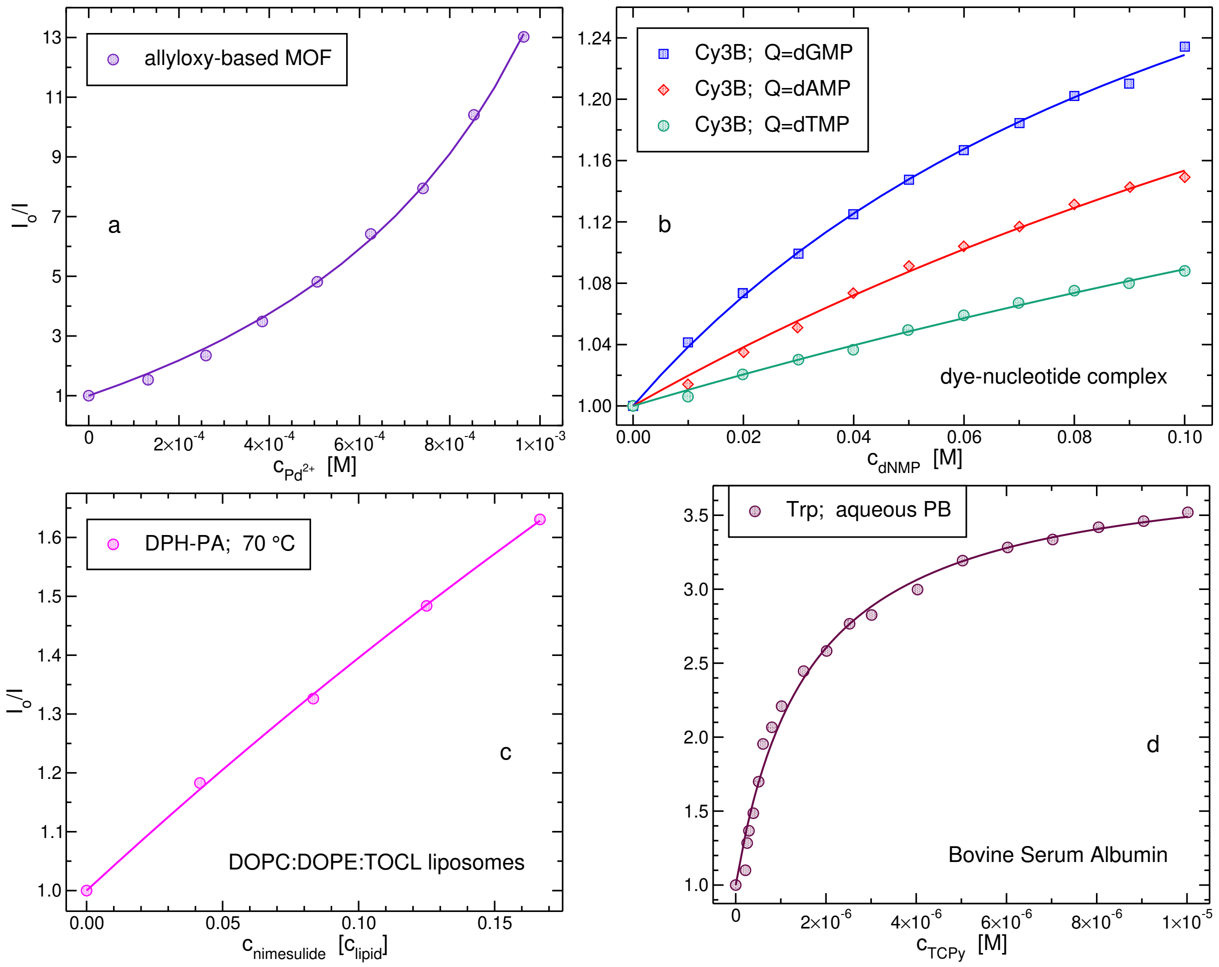

Figure S2.2: (a) Fitting of Eq. 44 (Eq. 43) to fluorescence of Al(III)-MOF based on 5-(allyloxy) isophthalic acid (H2AIA) ligand, quenched via static mechanism by Pd^{2+} (Ghosh et al.[66]). Data points were provided by the authors and the fitting results are shown in Table S2.1. (b)-(d) Fitting of Eq. 40 to fluorescence quenchings by a static mechanism that exhibit downward curvatures: (b) fluorescence of Cy3B dye quenched by three different 5'-deoxymononucleoside phosphates (Ranjit and Levitus[67]), (c) fluorescence of DPH-PA (3-(p-(6-phenyl)-1,3,5-hexatrienyl)phenylpropionic acid), incorporated in liposomes, quenched by 4-nitro-2-phenoxymethane-sulfonanilide, nimesulide (Monteiro et al.[68]), (d) fluorescence of tryptophan residues in bovine serum albumin, BSA, quenched by TCPy (3,5,6-trichloro-2-pyridinol) in aqueous phosphate buffer, PB, (Dahiya et al.[69]). Details of the fittings are given in Table S2.2.

Table S2.1: Results of fitting Eq. 44 (Eq. 43) to fluorescence quenching data shown in Fig. S2.1 and Fig. S2.2a, wherein the quenching mechanism is static and the fluorophore-quencher systems are subject to different types of confinement. α is the average fraction of excited fluorophore (Eq. 42) and its relation to $\mathcal{Z}_s$ is given in Eq. 41. The entries colored in gray are one-parameter fitting results in which K_{FQ}^{ss}, for all emission wavelengths, is fixed to its geometric average calculated by the two-parameter fitting. R is the correlation coefficient of the fitting.

Fluorophore	Quencher	plotted in	K_{FQ}^{ss}	$\mathcal{Z}_s$ (α)	R
OFX 293 $°C$ (β-cyclodextrin)[64]	Cu^{2+}	Fig. S2.1a	$1.00 \cdot 10^5$	1.32 (0.242)	0.9986
OFX 303 $°C$ (β-cyclodextrin)[64]	Cu^{2+}	Fig. S2.1a	$7.67 \cdot 10^4$	1.46 (0.316)	0.9989
OFX 313 $°C$ (β-cyclodextrin)[64]	Cu^{2+}	Fig. S2.1a	$6.38 \cdot 10^4$	1.57 (0.362)	0.9986
NSCDs (NPs; ethanol)[42]	CT	Fig. S2.1b	18.5	1.35 (0.261)	0.9943
pSiNPs (850 nm; 4 °C)[32]	BV^{2+}	Fig. S2.1c	$2.22 \cdot 10^6$	1.36 (0.262)	0.9977
pSiNPs (850 nm; 4 °C)[32]	BV^{2+}	Fig. S2.1c	$1.31 \cdot 10^6$	1.74 (0.427)	0.9908
pSiNPs (800 nm; 4 °C)[32]	BV^{2+}	Fig. S2.1c	$1.25 \cdot 10^6$	1.56 (0.359)	0.9971
pSiNPs (800 nm; 4 °C)[32]	BV^{2+}	Fig. S2.1c	$1.31 \cdot 10^6$	1.52 (0.342)	0.9973
pSiNPs (750 nm; 4 °C)[32]	BV^{2+}	Fig. S2.1c	$8.03 \cdot 10^5$	1.66 (0.399)	0.9964
pSiNPs (750 nm; 4 °C)[32]	BV^{2+}	Fig. S2.1c	$1.31 \cdot 10^6$	1.23 (0.184)	0.9957
GBA (vesicles)[65]	Cu^{2+}	Fig. S2.1d	1830	1.44 (0.303)	0.9910
allyloxy-based MOF[66]	Pd^{2+}	Fig. S2.2a	4580	1.13 (0.117)	0.9994

Table S2.2: Results of fitting fluorescence quenching by static mechanism that exhibit downward curvature (shown in Fig. S2.2) with Eq. 40. As indicated in the text, $\mathcal{W} = \beta - \alpha$, where α and β are populations' coefficients defined in Eq. 39.

Fluorophore	Quencher	plotted in	$\mathcal{Z}_s K_{FQ}^{ss}$	$\mathcal{W}$	R
Cy3B[67]	dGMP	Fig. S2.2b	4.17	1.97	0.9993
Cy3B[67]	dAMP	Fig. S2.2b	2.04	1.61	0.9983
Cy3B[67]	dTMP	Fig. S2.2b	1.07	1.83	0.9980
DPH-PA (70°C)[68]	nimesulide	Fig. S2.2c	4.27	0.185	0.9997
tryptophan; BSA (aqueous PB)[69]	TCPy	Fig. S2.2d	$1.80 \cdot 10^6$	0.346	0.9953

SI-3 Testing Single-Fluorophore Quenching Model: Combined Mechanisms

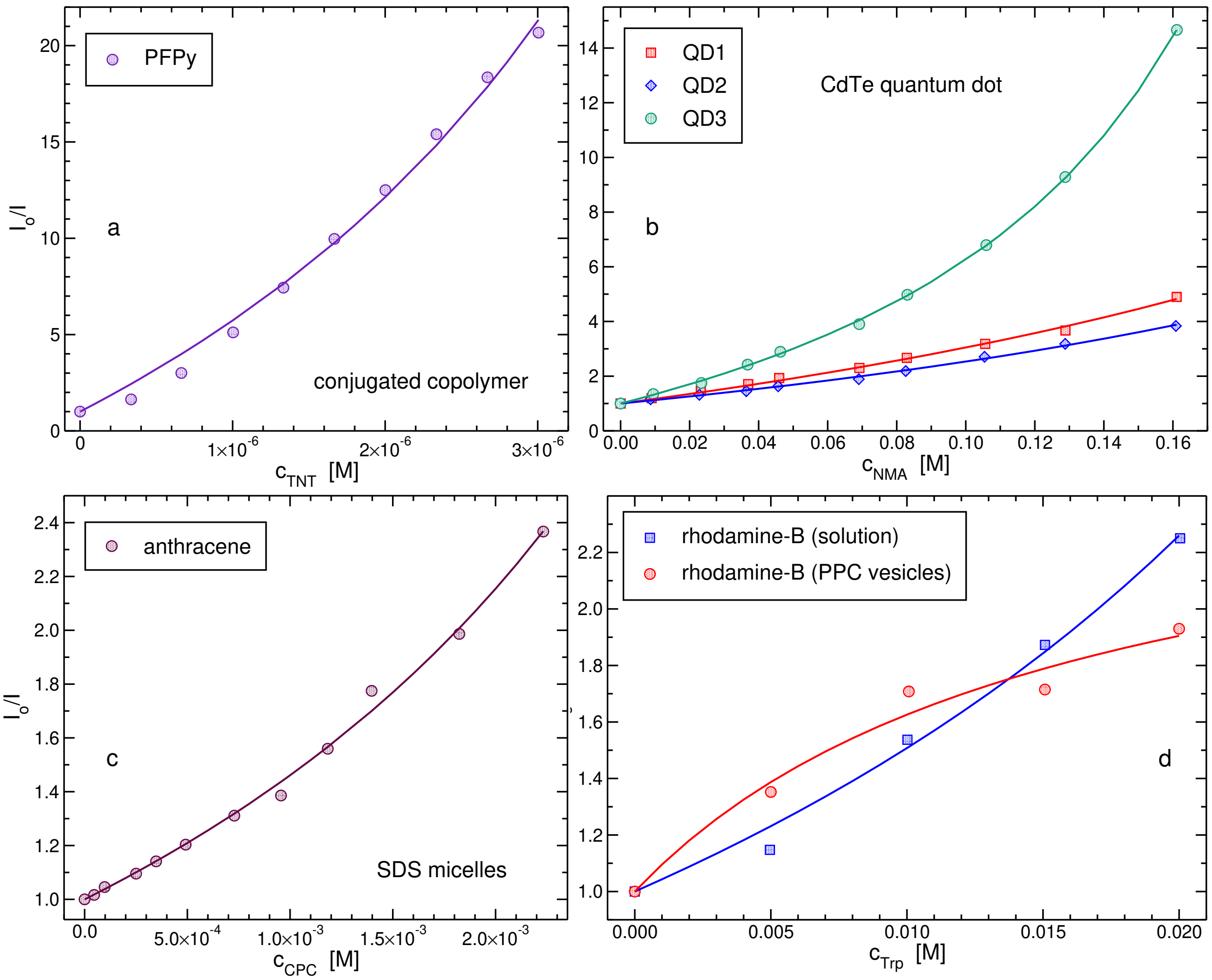

Figure S3.1: Fitting Eq. 44 to SV-plots governed by a combination of dynamic and static quenching mechanisms under different types of confinement. Fluorescence of (a) cationic pyridinium conjugated polymers, PFPy, quenched by trinitrotoluene, TNT (Tanwar et al.[29]); (b) CdTe quantum dot quenched by N-methylaniline, NMA (Bharadwaj et al.[70]); (c) anthracene in SDS micelles quenched by cetylpyridinium chloride, CPC (Soemo and Pemberton[28]); and (d) rhodamine-B, either in solution or in POPC:POPG:Cholesterol vesicles, quenched by tryptophan, Trp (Dey et al.[71]). Details of the fittings results are given in Table S3.1.

Table S3.1: Results of fitting Eq. 44 to fluorescence quenching data shown in Fig. S3.1 wherein the quenching is reported to take place by a combination of dynamic and static mechanisms.

Fluorophore	Quencher	plotted in	K	$\mathcal{Z}$	R
PFPy (cationic conjugated polymers)[29]	TNT	Fig. S3.1a	$3.98 \cdot 10^6$	1.03	0.9972
QD1 (CdTe quantum dot)[70]	NMA	Fig. S3.1b	14.9	1.12	0.9985
QD2 (CdTe quantum dot)[70]	NMA	Fig. S3.1b	10.5	1.18	0.9984
QD3 (CdTe quantum dot)[70]	NMA	Fig. S3.1b	28.5	1.13	0.9999
anthracene (SDS)[28]	CPC	Fig. S3.1c	217	1.77	0.9979
rhodamine-B (solution)[71]	Trp	Fig. S3.1d	26.2	1.62	0.9965
rhodamine-B (PPC vesicles)[71]	Trp	Fig. S3.1d	164	0.620	0.9871

SI-4 Confinement within Micellar Structures

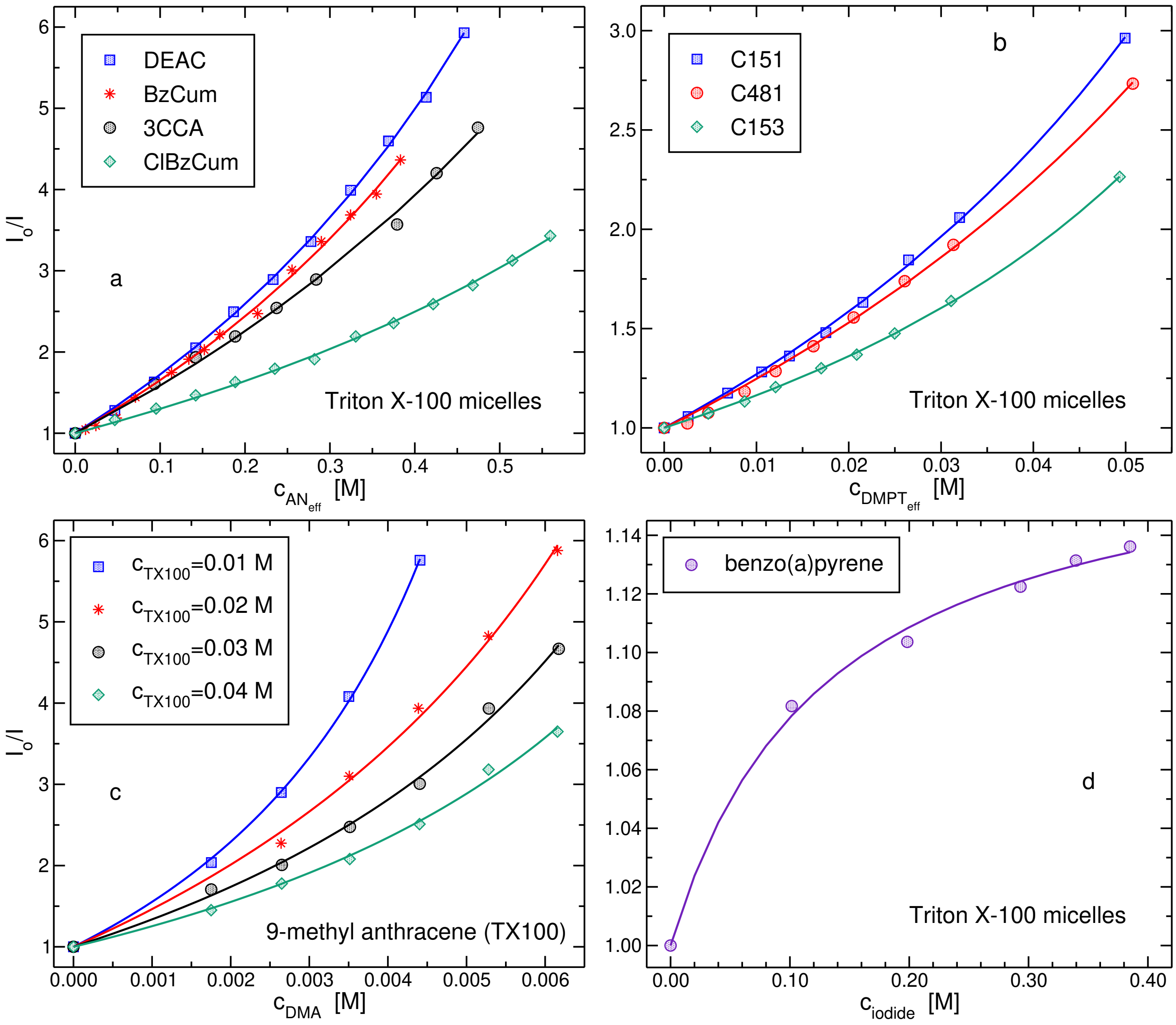

Figure S4.1: Fitting Eq. 44 to fluorescence quenching where fluorophores are encapsulated inside Triton X-100 micelles. Coumarin derivatives quenched by (a) aniline, AN (Tablet et al.[72]), and (b) N,N-dimethyl-p-toluidine, DMPT (Kumbhakar et al.[73]). (c) 9-methyl anthracene quenched by N,N-dimethylaniline, DMA, at different concentrations of TX100 (Blatt et al.[74]). (d) Benzo(a)pyrene quenched by iodide (Cramb and Beck[75]). Quencher's concentrations in (a) are effective values within the micelles, whereas in other panels (b-d), they correspond to the concentrations introduced into the whole system. Details of the fittings results are given in Table S4.1.

Table S4.1: Results of fitting Eq. 44 to fluorescence quenching data shown in Fig. S4.1, where fluorophore and quencher are confined (for the duration of the measurements) to assemblies formed by the surfactant Triton X-100.

Fluorophore	Quencher	plotted in	K	$\mathcal{Z}$	R
DEAC (TX100)[72]	aniline	Fig. S4.1a	5.76	1.15	0.9997
BzCum (TX100)[72]	aniline	Fig. S4.1a	5.17	1.16	0.9989
3CCA (TX100)[72]	aniline	Fig. S4.1a	4.88	1.13	0.9985
ClBzCum (TX100)[72]	aniline	Fig. S4.1a	2.19	1.28	0.9995
C151 (TX100)[73]	DMPT	Fig. S4.1b	17.7	1.41	0.9998
C481 (TX100)[73]	DMPT	Fig. S4.1b	16.7	1.39	0.9994
C153 (TX100)[73]	DMPT	Fig. S4.1b	6.61	2.27	0.9999
9-methyl anthracene (TX100: $0.01\ M$)[74]	DMA	Fig. S4.1c	358	1.35	0.9998
9-methyl anthracene (TX100: $0.02\ M$)[74]	DMA	Fig. S4.1c	354	1.21	0.9983
9-methyl anthracene (TX100: $0.03\ M$)[74]	DMA	Fig. S4.1c	233	1.34	0.9983
9-methyl anthracene (TX100: $0.04\ M$)[74]	DMA	Fig. S4.1c	159	1.48	0.9981
benzo(a)pyrene (TX100)[75]	iodide	Fig. S4.1d	8.98	0.153	0.9984

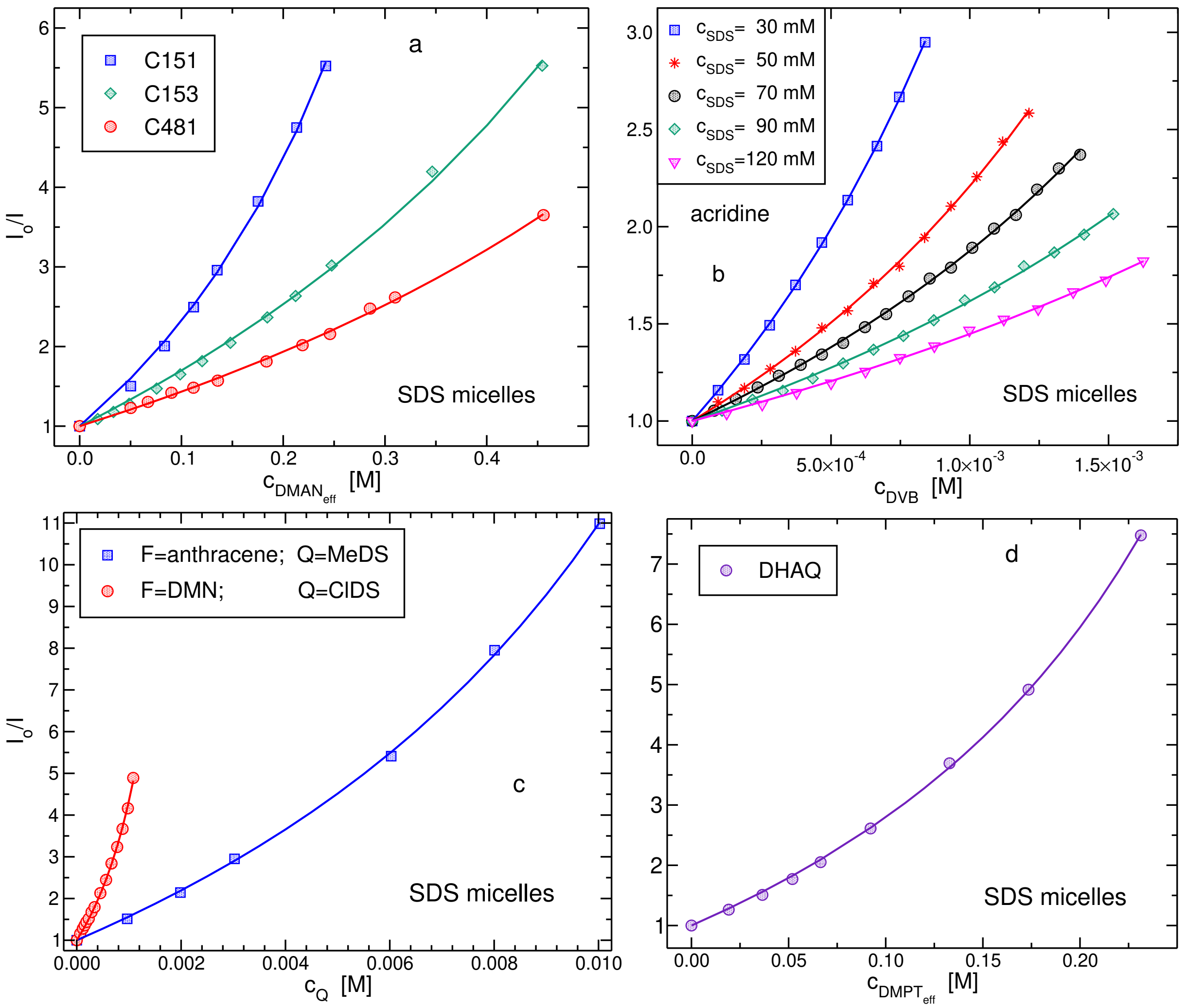

Figure S4.2: Fitting Eq. 44 to fluorescence quenching where fluorophores are encapsulated inside sodium dodecyl sulfate (SDS) micelles. Fluorescence quenching of (a) coumarin derivatives by dimethyl aniline, DMAN (Kumbhakar et al.[76]); (b) acridine by divinyl benzene, DVB, at five different SDS concentrations (Buchviser and Gehlen[59]); (c) anthracene by p-methylbenzene diazonium tetrafluoroborate, MeDS, as well as 1,4-dimethylnaphthalene, DMN, by p-chlorobenzene diazonium tetrafluoroborate, ClDS (Timpe et al.[77]); (d) anthraquinone (DHAQ) dye by N,N-dimethyl-p-toluidine, DMPT (Kumbhakar et al.[78]). Details of the fittings results are given in Table S4.2.

Table S4.2: Results of fitting Eq. 44 to fluorescence quenching data shown in Fig. S4.2, where fluorophore and quencher are confined within SDS micelles.

Fluorophore	Quencher	plotted in	K	$\mathcal{Z}$	R
C151 (SDS)[76]	DMAN	Fig. S4.2a	9.19	1.19	0.9995
C153 (SDS)[76]	DMAN	Fig. S4.2a	5.65	1.14	0.9995
C481 (SDS)[76]	DMAN	Fig. S4.2a	3.36	1.20	0.9993
acridine (SDS: 30 mM)[59]	DVB	Fig. S4.2b	1250	1.29	0.9999
acridine (SDS: 50 mM)[59]	DVB	Fig. S4.2b	589	1.48	0.9997
acridine (SDS: 70 mM)[59]	DVB	Fig. S4.2b	435	1.54	0.9996
acridine (SDS: 90 mM)[59]	DVB	Fig. S4.2b	296	1.67	0.9996
acridine (SDS: 120 mM)[59]	DVB	Fig. S4.2b	224	1.69	0.9995
anthracene (SDS)[77]	MeDS	Fig. S4.2c	497	1.09	0.9998
DMN (SDS)[77]	ClDS	Fig. S4.2c	1610	1.25	0.9997
DHAQ (SDS)[78]	DMPT	Fig. S4.2d	12.0	1.18	0.9999

SI-5 Embedment in Vesicles'/Liposomes' Membrane

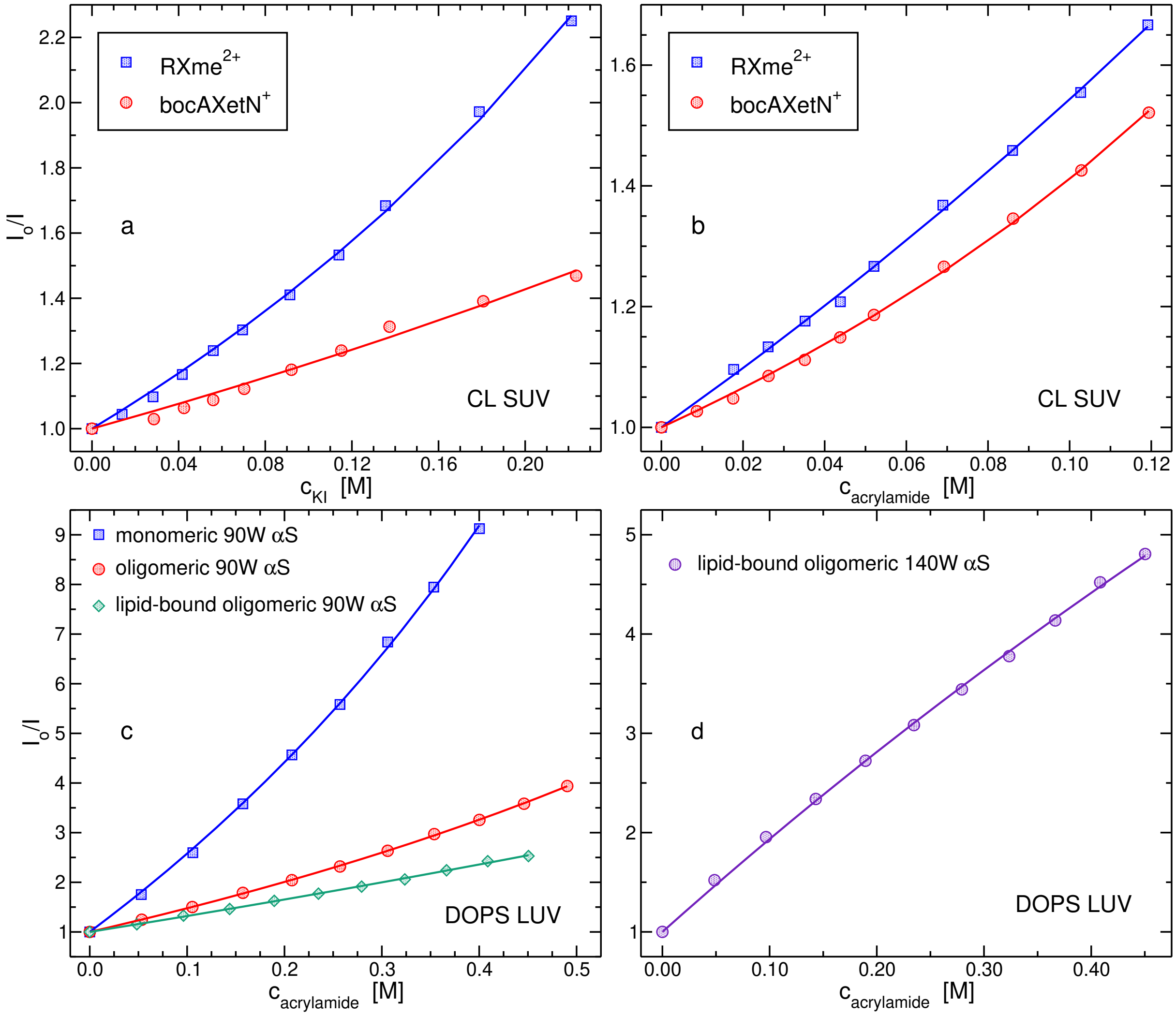

Figure S5.1: Fitting Eq. 44 to fluorescence quenching of tryptophan bound to lipid membranes. In data reported by de Kroon et al.[79], (a) and (b), the tryptophan residue is part of $RXme^{2+}$, or $bocAXetN^{+}$, pentapeptide bound to cardiolipin small unilamellar vesicles (CL SUV) and is quenched by (a) iodide and (b) acrylamide. In data reported by van Rooijen et al.[80], (c) and (d), the tryptophan residue is introduced in two single-mutant proteins of α-Synuclein (αS), (a) 90W and (b) 140W, bound to 1,2-dioleoyl phosphatidylserine large unilamellar vesicles (DOPS LUV) and is quenched by acrylamide. In (a), the results of fluorescence quenching of monomeric and oligomeric 90W αS in the absence of vesicles are presented for reference. Details of the fittings results are given in Table S5.1.

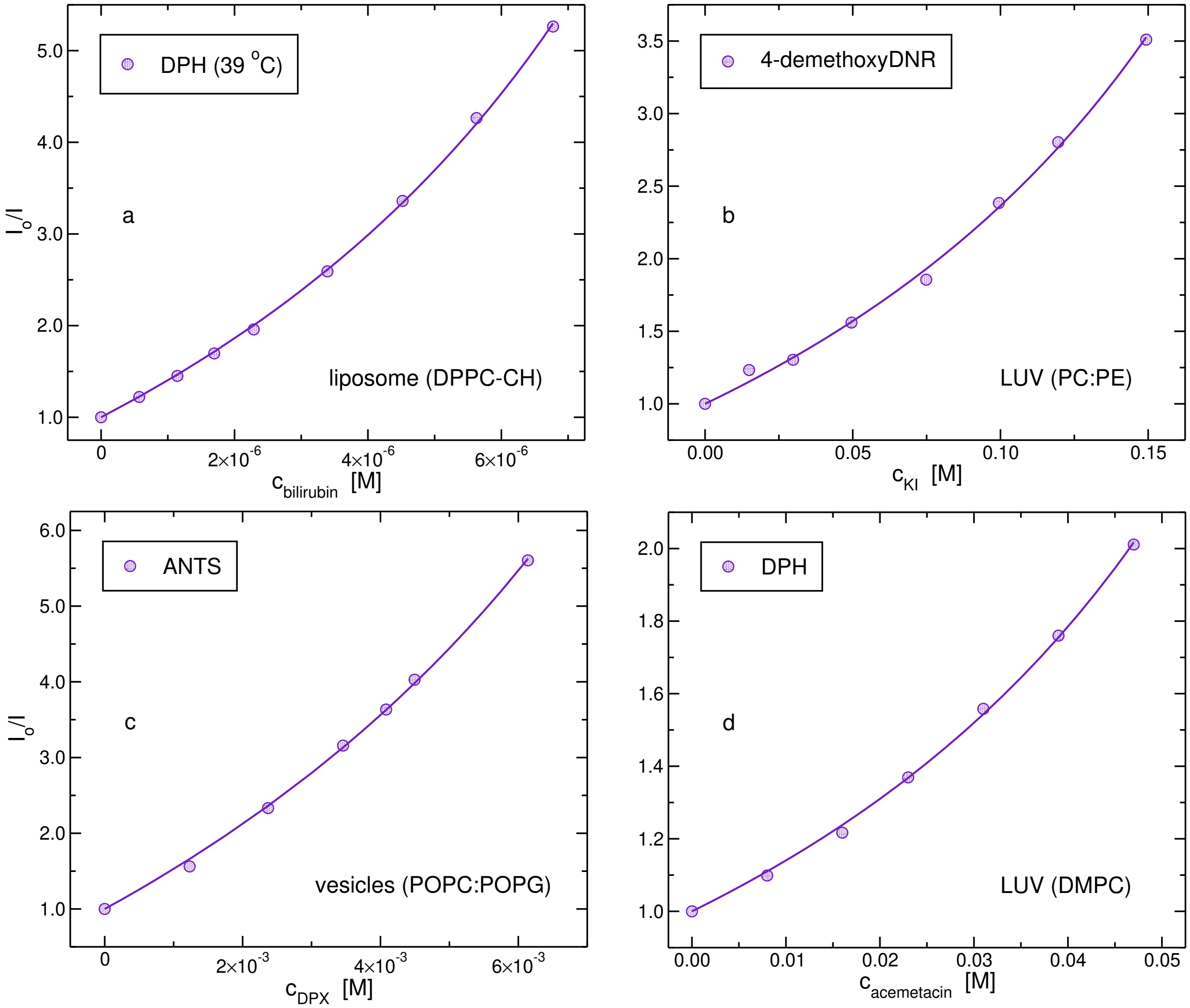

Figure S5.2: Fitting Eq. 44 to SV-plots plots of membrane-bound fluorophores: (a) 1,6-diphenyl-1,3,5-hexatriene (DPH), bound to DPPC bilayer containing cholesterol, by bilirubin (Nagaoka and Cowger[81]); (b) Anthracycline (4-demethoxydaunorubicin), bound to PC:PE LUV, by iodide at constant halide concentration (Praet et al.[82]); (c) 8-aminonapthalene-1,3,6 trisulfonic acid (ANTS), bound to vesicles of POPC:POPG, by p-xylene-bis-pyridinium bromide (DPX) (Ladokhin et al.[83]); and (d) DPH, bound to LUV of DMPC, by acemetacin (Lúcio et al.[84]). Results of the fittings are given in Table S5.1.

Table S5.1: Results of fitting Eq. 44 to fluorescence quenching of fluorophores confined to vesicles' membranes shown in Fig. S5.1 and Fig. S5.2.

Fluorophore	Quencher	plotted in	K	$\mathcal{Z}$	R
$RXme^{2+}$ (CL SUV)[79]	iodide	Fig. S5.1a	2.68	1.50	0.9995
$bocAXetN^{+}$ (CL SUV)[79]	iodide	Fig. S5.1a	1.23	1.52	0.9945
$RXme^{2+}$ (CL SUV)[79]	acrylamide	Fig. S5.1b	3.64	1.32	0.9995
$bocAXetN^{+}$ (CL SUV)[79]	acrylamide	Fig. S5.1b	0.660	4.71	0.9996
monomeric 90W αS (solution)[80]	acrylamide	Fig. S5.1c	13.9	1.05	0.9998
oligomeric 90W αS (solution)[80]	acrylamide	Fig. S5.1c	4.04	1.12	0.9999
oligomeric 90W αS (DOPS LUV)[80]	acrylamide	Fig. S5.1c	2.96	1.06	0.9995
oligomeric 140W αS (DOPS LUV)[80]	acrylamide	Fig. S5.1d	9.98	0.967	0.9997
DPH (liposome: DPPC-CH)[81]	bilirubin	Fig. S5.2a	$3.20 \cdot 10^5$	1.19	0.9998
4-demethoxyDNR (PC:PE LUV)[82]	iodide	Fig. S5.2b	6.98	1.40	0.9987
ANTS (vesicles: POPC:POPG)[83]	DPX	Fig. S5.2c	445	1.12	0.9996
DPH (DMPC LUV)[84]	acemetacin	Fig. S5.2d	4.13	3.10	0.9995

SI-6 Conjugated Polymer and Block Copolymer Systems

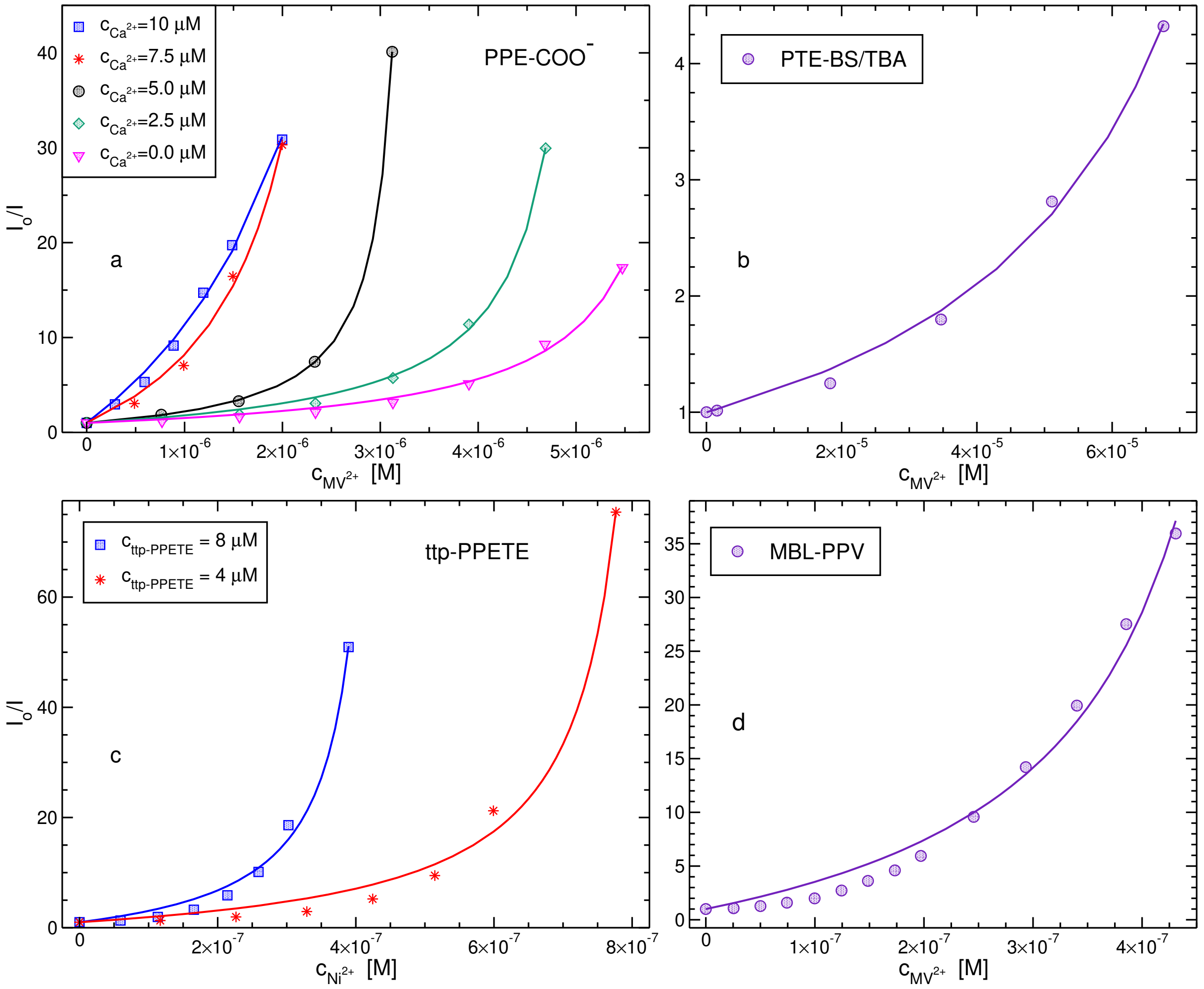

Figure S6.1: Fitting Eq. 44 to fluorescence quenching in conjugated polymer systems. (a) Quenching of poly(phenylene ethynylene), PPE-COO$^-$, by N,N'-dimethyl viologen, MV^{2+}, in methanol for different degrees of polymer's aggregation, which is controlled by the concentration Ca^{2+} (Jiang et al.[85]). (b) Quenching of poly[2-(3-thienyl)ethyloxy-4-butylsulfonate)], PTE-BS, in the presence of the surfactant tetrabutylammonium perchlorate, TBA, by MV^{2+} (López-Cabarcos et al.[86]). (c) Quenching of poly-2,5-didodecyloxyphenylene ethynylene 3-trans-(((4'-p-phenyl)-2,2':6,2"-terpyridine)-vinylene)-thiophene ethynylene, ttp-PPETE, by Ni^{2+} (Murphy et al.[87]). (d) Quenching of poly[5-methoxy-2-(4-sulfobutoxy)-1,4-phenylenevinylene], MBL-PPV, by MV^{2+} (Wang et al.[43]). Fitting results are summarized in Table S6.1.

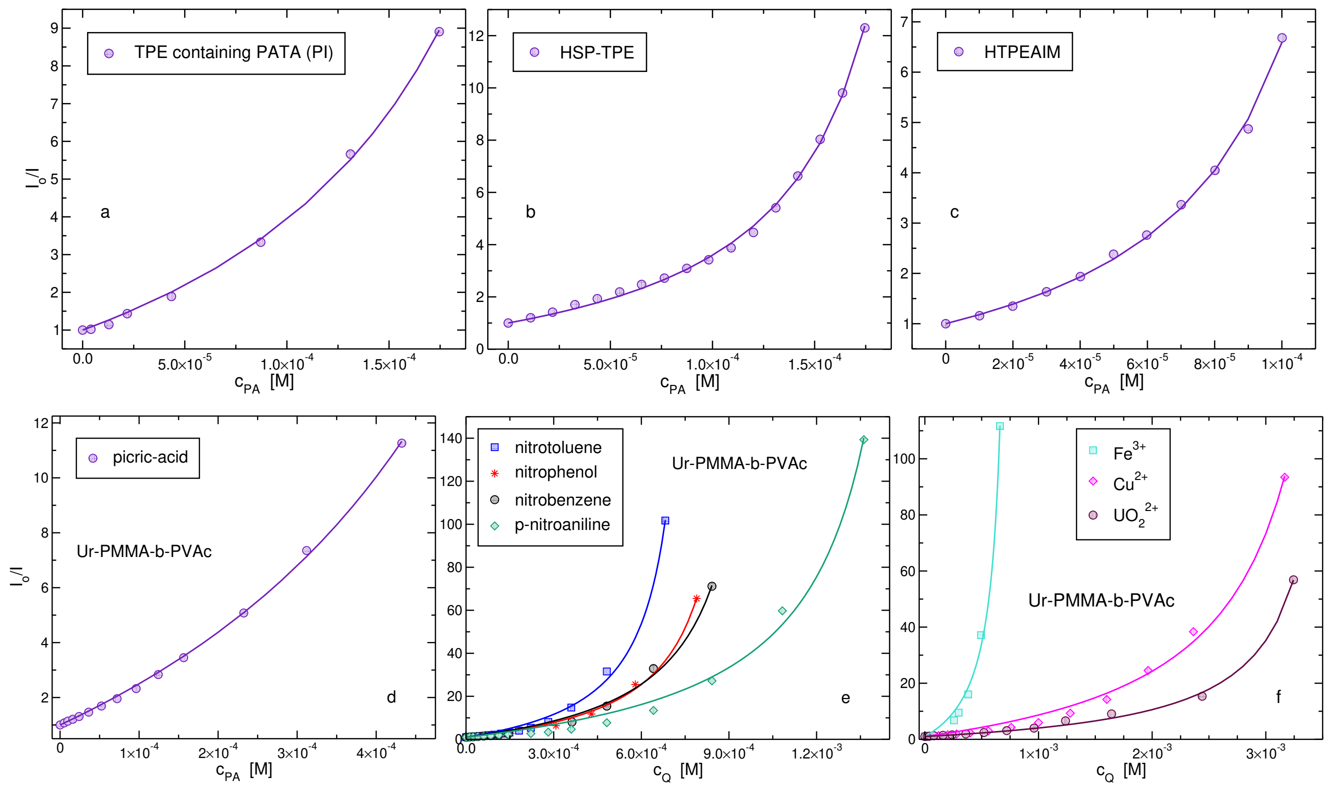

Figure S6.2: Upper panel: Fitting Eq. 44 to fluorescence, quenched by picric acid, of different tetraphenylethene, TPE, polymers. (a) TPE, containing poly(aroyltriazole), PATA (Qin et al.[88]); (b) poly(3-ethyl-3-oxetanemethanol)-star-poly(ethylene oxide), HSP, copolymers containing TPE derivative (2-(4-vinylphenyl)ethene-1,1,2-triyl)tribenzene (Nabeel et al.[89]); (c) TPE-functionalized arylimidazole derivative, HTPEAIM (Yin et al.[90]). Lower panel: Quenching of uracil-poly(methyl methacrylate)-block-poly(vinyl acetate) block copolymer, Ur-PMMA-b-PVAc, by (d) picric acid; (e) several nitro-aromatic compounds; (f) metal ions (Podasca et al.[91]). Fitting results are summarized in Table S6.1.

Table S6.1: Results of fitting Eq. 44 to fluorescence quenching data of conjugated polymer and block copolymer systems shown in Fig. S6.1 and Fig. S6.2.

Fluorophore	Quencher	plotted in	K	$\mathcal{Z}$	R
PPE-COO$^-$ (10.0 μM $CaCl_2$)[85]	MV^{2+}	Fig. S6.1a	$7.54 \cdot 10^6$	1.03	0.9983
PPE-COO$^-$ (7.5 μM $CaCl_2$)[85]	MV^{2+}	Fig. S6.1a	$4.41 \cdot 10^6$	1.08	0.9979
PPE-COO$^-$ (5.0 μM $CaCl_2$)[85]	MV^{2+}	Fig. S6.1a	$5.34 \cdot 10^5$	1.56	0.9999
PPE-COO$^-$ (2.5 μM $CaCl_2$)[85]	MV^{2+}	Fig. S6.1a	$4.48 \cdot 10^5$	1.43	0.9994
PPE-COO$^-$ (0.0 μM $CaCl_2$)[85]	MV^{2+}	Fig. S6.1a	$2.77 \cdot 10^5$	1.56	0.9980
PTE-BS/TBA[86]	MV^{2+}	Fig. S6.1b	$6.80 \cdot 10^3$	2.44	0.9983
ttp-PPETE ($8\ \mu M$)[87]	Ni^{2+}	Fig. S6.1c	$1.36 \cdot 10^7$	1.17	0.9967
ttp-PPETE ($4\ \mu M$)[87]	Ni^{2+}	Fig. S6.1c	$6.85 \cdot 10^6$	1.17	0.9968
MBL-PPV (TBA)[43]	MV^{2+}	Fig. S6.1d	$1.92 \cdot 10^7$	1.09	0.9954
TPE containing PATA (PI)[88]	picric acid	Fig. S6.2a	$1.66 \cdot 10^4$	1.19	0.9996
HSP-TPE copolymer[89]	picric acid	Fig. S6.2b	$9.88 \cdot 10^3$	1.45	0.9993
HTPEAIM[90]	picric acid	Fig. S6.2c	$9.69 \cdot 10^3$	1.72	0.9990
Ur-PMMA-b-PVAc[91]	picric acid	Fig. S6.2d	$1.25 \cdot 10^4$	1.08	0.9997
Ur-PMMA-b-PVAc[91]	nitrotoluene	Fig. S6.2e	$2.09 \cdot 10^4$	1.06	0.9984
Ur-PMMA-b-PVAc[91]	nitrophenol	Fig. S6.2e	$1.48 \cdot 10^4$	1.07	0.9988
Ur-PMMA-b-PVAc[91]	nitrobenzene	Fig. S6.2e	$1.72 \cdot 10^4$	1.05	0.9980
Ur-PMMA-b-PVAc[91]	p-nitroaniline	Fig. S6.2e	$1.53 \cdot 10^3$	1.04	0.9977
Ur-PMMA-b-PVAc[91]	Fe^{3+}	Fig. S6.2f	$2.18 \cdot 10^4$	1.06	0.9984
Ur-PMMA-b-PVAc[91]	Cu^{2+}	Fig. S6.2f	$5.47 \cdot 10^3$	1.05	0.9980
Ur-PMMA-b-PVAc[91]	$UO_2{}^{2+}$	Fig. S6.2f	$1.95 \cdot 10^3$	1.14	0.9989

SI-7 Metal Organic Frameworks

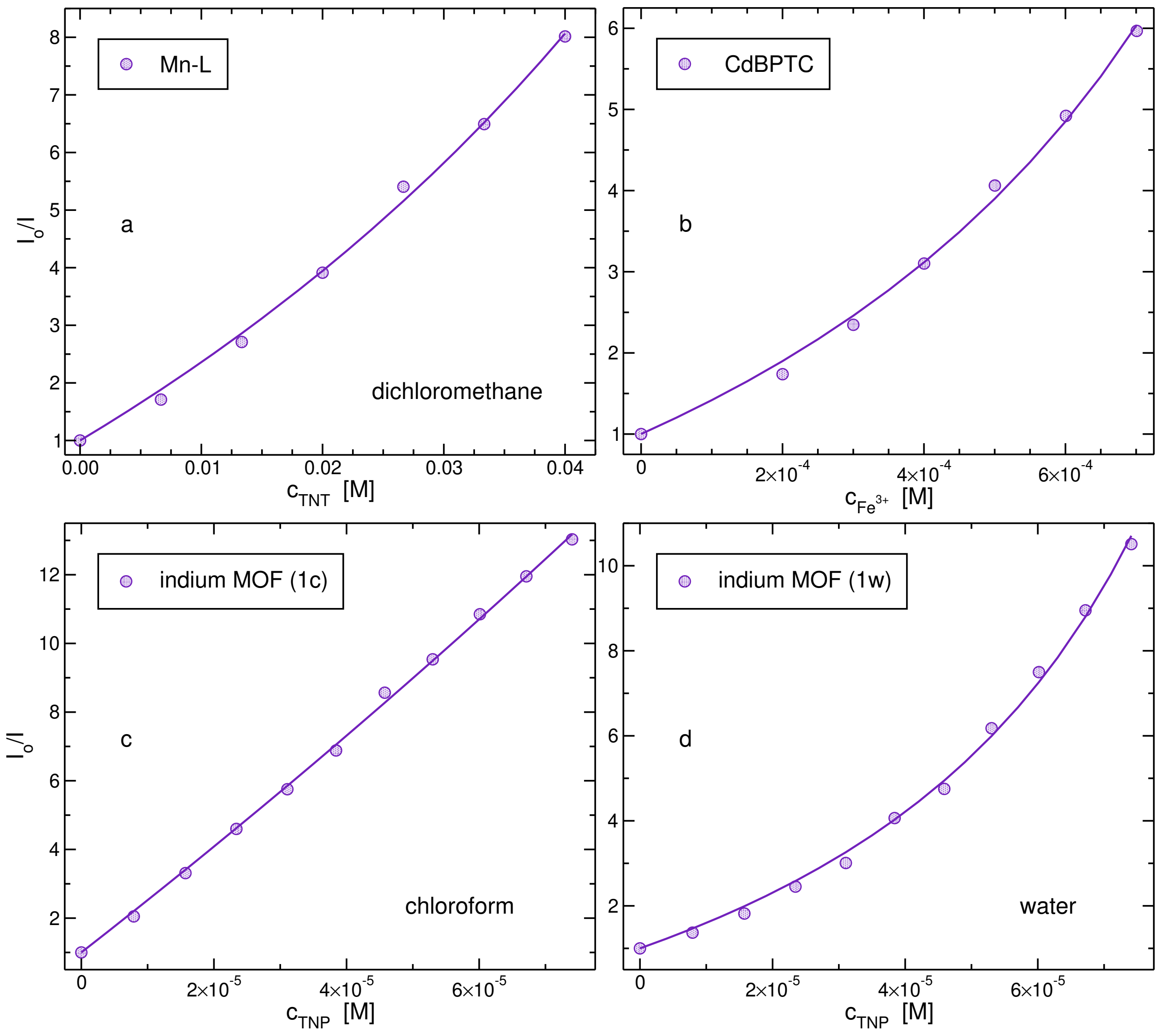

Figure S7.1: Fitting Eq. 44 to fluorescence quenching in metal organic frameworks. (a) Mn^{2+}, liganded by pyrene tetra-carboxylate, MOF quenched by 2,4,6-trinitrotoluene, TNT (Bajpai et al.[92]); (b) Cd^{2+}, liganded by biphenyltetracarboxylate, CdBPTC, MOF quenched by Fe^{3+} (Xu et al.[93]); In^{3+}, liganded by hydroxide and 2,5-dihydoxy-1,4-benzenedicarboxylate (H_2DOBDC), MOF quenched by 2,4-dinitrophenol, TNP, in (c) chloroform and (d) water (Sharma et al.[94]). Results of the fittings are given in Table S7.1.

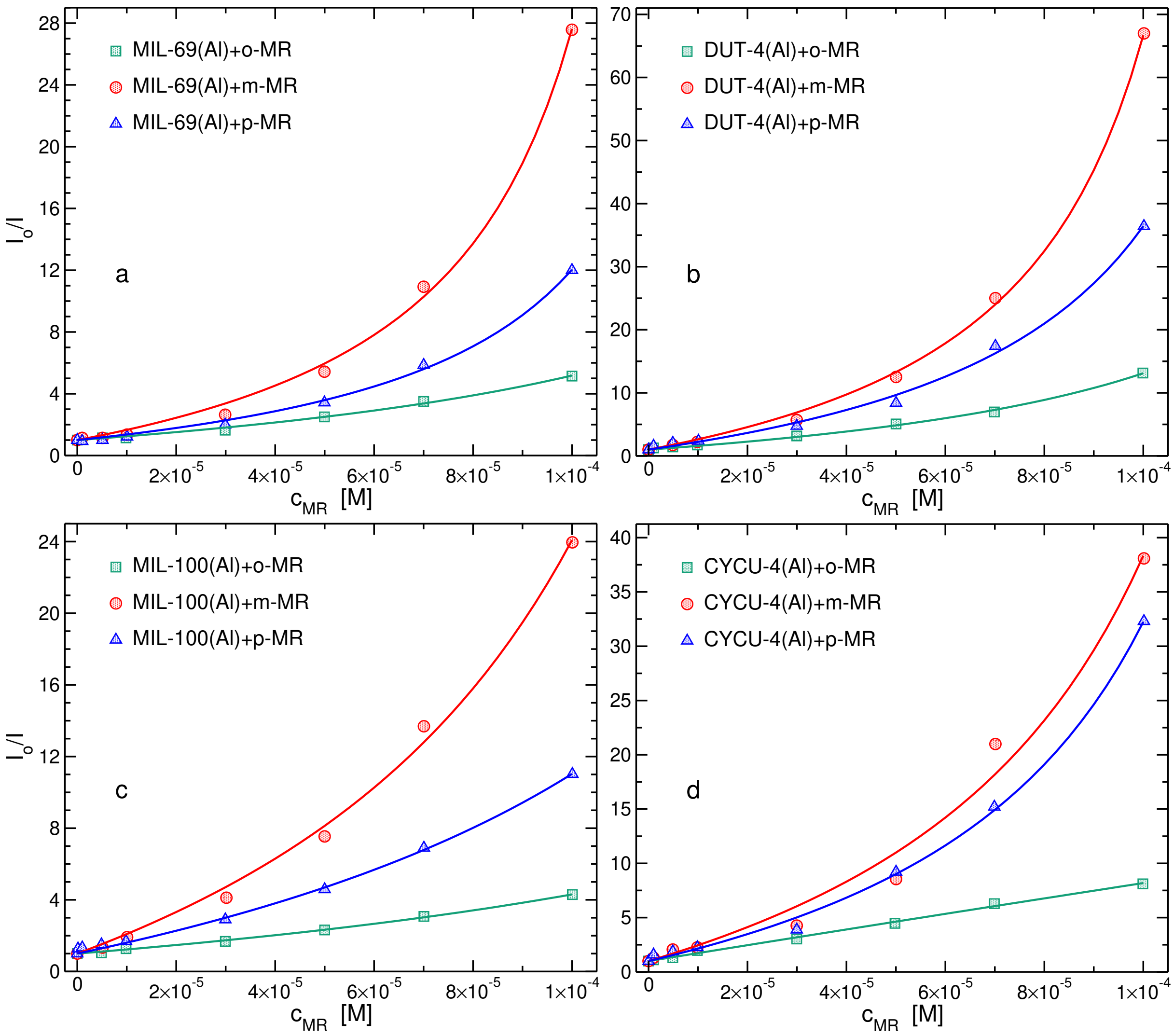

Figure S7.2: Fitting Eq. 44 to fluorescence quenching of Al-based metal-organic frameworks by ortho-, meta-, and para-, Methyl Red (MR) quenchers reported by Chen et al.[95]. In (a) MIL-69 and (b) DUT-4, the Al^{3+} is liganded by hydroxide and 2,6-naphthalenedicarboxylates. In (c) MIL-100, the Al^{3+} is liganded by fluoride, water, and 1,3,5-benzenetricarboxylate, and in (d) CYCU-4, the Al^{3+} is liganded by hydroxide and 4,4-stilbenedicarboxylate. Results of the fittings are given in Table S7.1.

Table S7.1: Results of Fitting Eq. 44 to fluorescence quenching inside metal-organic frameworks (Fig. S7.1 and Fig. S7.2).

Fluorophore	Quencher	plotted in	K	$\mathscr{Z}$	R
Mn-L[92]	TNT	Fig. S7.1a	119	1.06	0.9987
CdBPTC[93]	Fe^{3+}	Fig. S7.1b	3260	1.20	0.9983
In(OH)(H_2DOBDC) chloroform (1c)[94]	TNP	Fig. S7.1c	$1.50 \cdot 10^5$	1.01	0.9995
In(OH)(H_2DOBDC) water (1w)[94]	TNP	Fig. S7.1d	$4.74 \cdot 10^4$	1.16	0.9987
MIL-69(Al)[95]	o-MR	Fig. S7.2a	$1.91 \cdot 10^4$	1.23	0.9983
MIL-69(Al)[95]	m-MR	Fig. S7.2a	$5.33 \cdot 10^4$	1.14	0.9990
MIL-69(Al)[95]	p-MR	Fig. S7.2a	$2.68 \cdot 10^4$	1.26	0.9992
DUT-4(Al)[95]	o-MR	Fig. S7.2b	$5.10 \cdot 10^4$	1.10	0.9992
DUT-4(Al)[95]	m-MR	Fig. S7.2b	$14.3 \cdot 10^4$	1.05	0.9996
DUT-4(Al)[95]	p-MR	Fig. S7.2b	$10.8 \cdot 10^4$	1.06	0.9982
MIL-100(Al)[95]	o-MR	Fig. S7.2c	$1.91 \cdot 10^4$	1.17	0.9993
MIL-100(Al)[95]	m-MR	Fig. S7.2c	$9.76 \cdot 10^4$	1.06	0.9984
MIL-100(Al)[95]	p-MR	Fig. S7.2c	$5.42 \cdot 10^4$	1.08	0.9992
CYCU-4(Al)[95]	o-MR	Fig. S7.2d	$7.36 \cdot 10^4$	0.997	0.9983
CYCU-4(Al)[95]	m-MR	Fig. S7.2d	$13.0 \cdot 10^4$	1.05	0.9932
CYCU-4(Al)[95]	p-MR	Fig. S7.2d	$10.1 \cdot 10^4$	1.06	0.9989

SI-8 β-Cyclodextrin and Amylose Structures

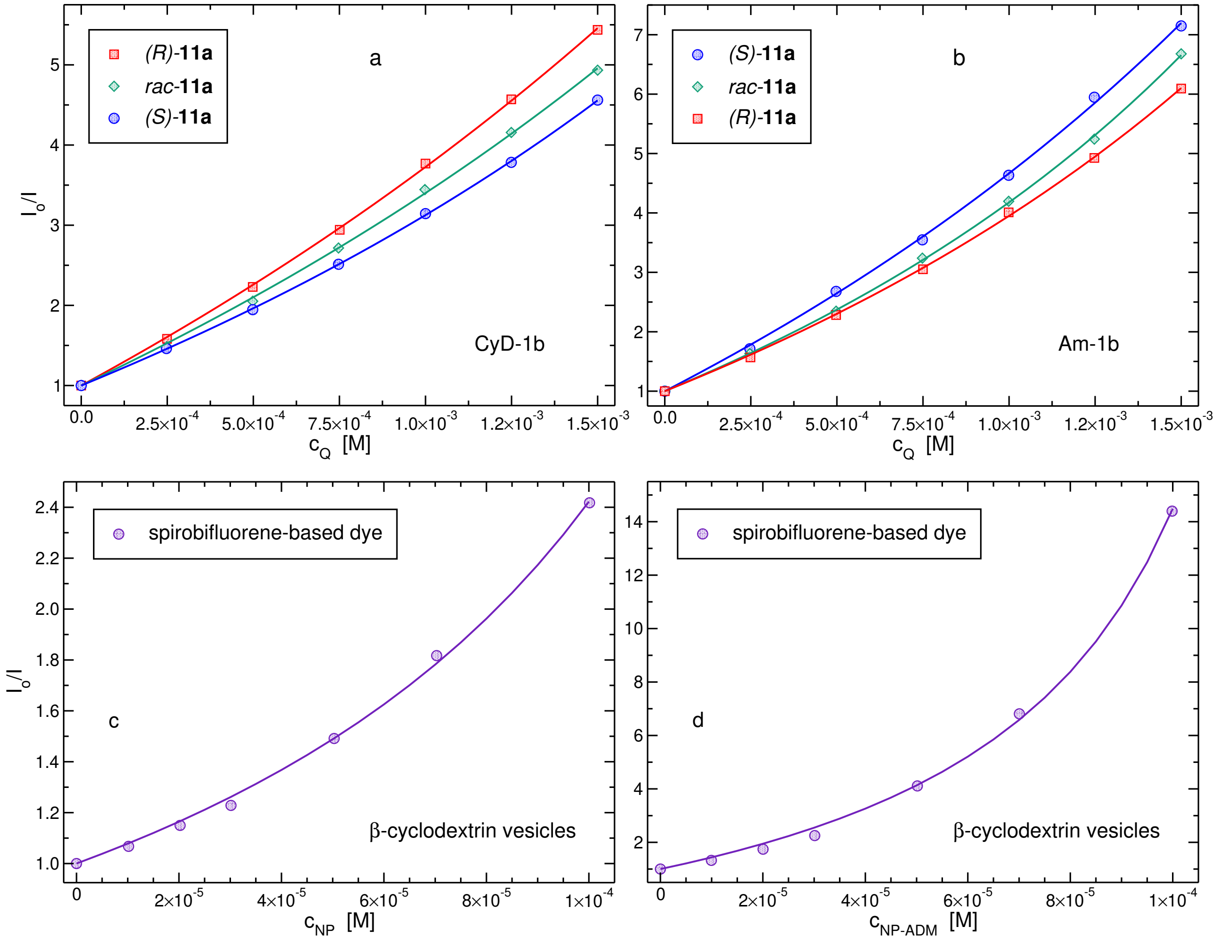

Figure S8.1: Fitting Eq. 44 to fluorescence quenching inside β-cyclodextrin and amylose structures. (a) Fluorescent derivative of (a) β-cyclodextrin, and (b) amylose, quenched by chiral (*R*, *S*, and *racemic* aromatic nitro compounds (Ikai et al.[96]). Quenching of spirobifluorene-based dye (2,7-bis-(4-(N,N-diphenylamino)phen-1-yl)-9,9-spirobifluorene) trapped inside β-cyclodextrin vesicles by (c) 4-nitrophenol, NP, and (d) 4-nitrophenol covalently connected to adamantane, NP-ADM (Schibilla et al.[97]). Results of the fittings are given in Table S8.1.

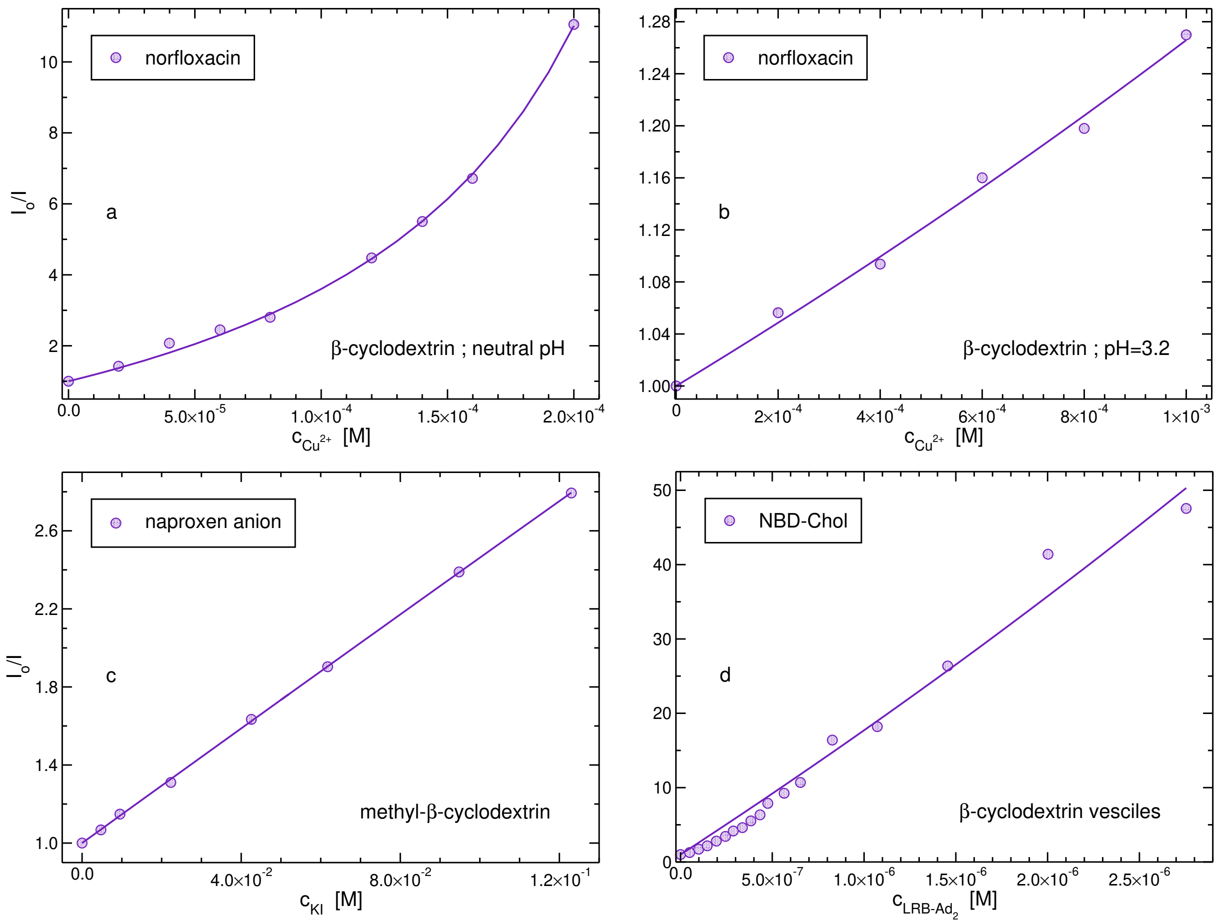

Figure S8.2: Fitting Eq. 44 to fluorescence quenching inside β-cyclodextrin. Quenching of norfloxacin by copper(II) under (a) neutral, and (b) acidic, pH (Padhan et al.[98]); (c) Quenching of naproxen anion, complexed with methyl-β-cyclodextrin, by iodide (Sadlej-Sosnowska and Siemiarczuk[99]); (d) Quenching of N-(7-nitrobenz-2-oxa-1,3-diazol-4-yl)-labeled cholesterol, NBD-Chol, by N-(lissamine-rhodamine B)-labeled divalent adamantyl, LRB-Ad_2, inside β-cyclodextrin vesicles (Lim et al.[100]). Results of the fittings are given in Table S8.1.

Table S8.1: Results of Fitting Eq. 44 to fluorescence quenching inside β-cyclodextrin (Fig. S8.1 and Fig. S8.2).

Fluorophore	Quencher	plotted in	K	$\mathcal{Z}$	R
CyD-1b[96]	aromatic-nitro mol. *(R)*-**11a**	Fig. S8.1a	$2.20 \cdot 10^3$	1.06	0.9999
CyD-1b[96]	aromatic-nitro mol. *rac*-**11a**	Fig. S8.1a	$1.88 \cdot 10^3$	1.08	0.9998
CyD-1b[96]	aromatic-nitro mol. *(S)*-**11a**	Fig. S8.1a	$1.59 \cdot 10^3$	1.11	1.000
Am-1b[96]	aromatic-nitro mol. *(S)*-**11a**	Fig. S8.1b	$2.81 \cdot 10^3$	1.07	0.9997
Am-1b[96]	aromatic-nitro mol. *rac*-**11a**	Fig. S8.1b	$2.18 \cdot 10^3$	1.11	0.9999
Am-1b[96]	aromatic-nitro mol. *(R)*-**11a**	Fig. S8.1b	$2.12 \cdot 10^3$	1.10	0.9998
spirobifluorene-based dye[97]	4-nitrophenol	Fig. S8.1c	$2.67 \cdot 10^3$	2.78	0.9993
spirobifluorene-based dye[97]	4-nitrophenol-adamantane	Fig. S8.1d	$33.8 \cdot 10^3$	1.21	0.9995
norfloxacin; neutral pH[98]	Cu^{2+}	Fig. S8.2a	$1.43 \cdot 10^4$	1.23	0.9994
norfloxacin; pH=3.2[98]	Cu^{2+}	Fig. S8.2b	$1.34 \cdot 10^2$	1.78	0.9974
naproxen anion[99]	KI	Fig. S8.2c	$1.48 \cdot 10^1$	0.995	0.9999
NBD-Chol[100]	LRB-Ad_2	Fig. S8.2d	$1.61 \cdot 10^7$	1.00	0.9918

SI-9 Enclosure in Microemulsions

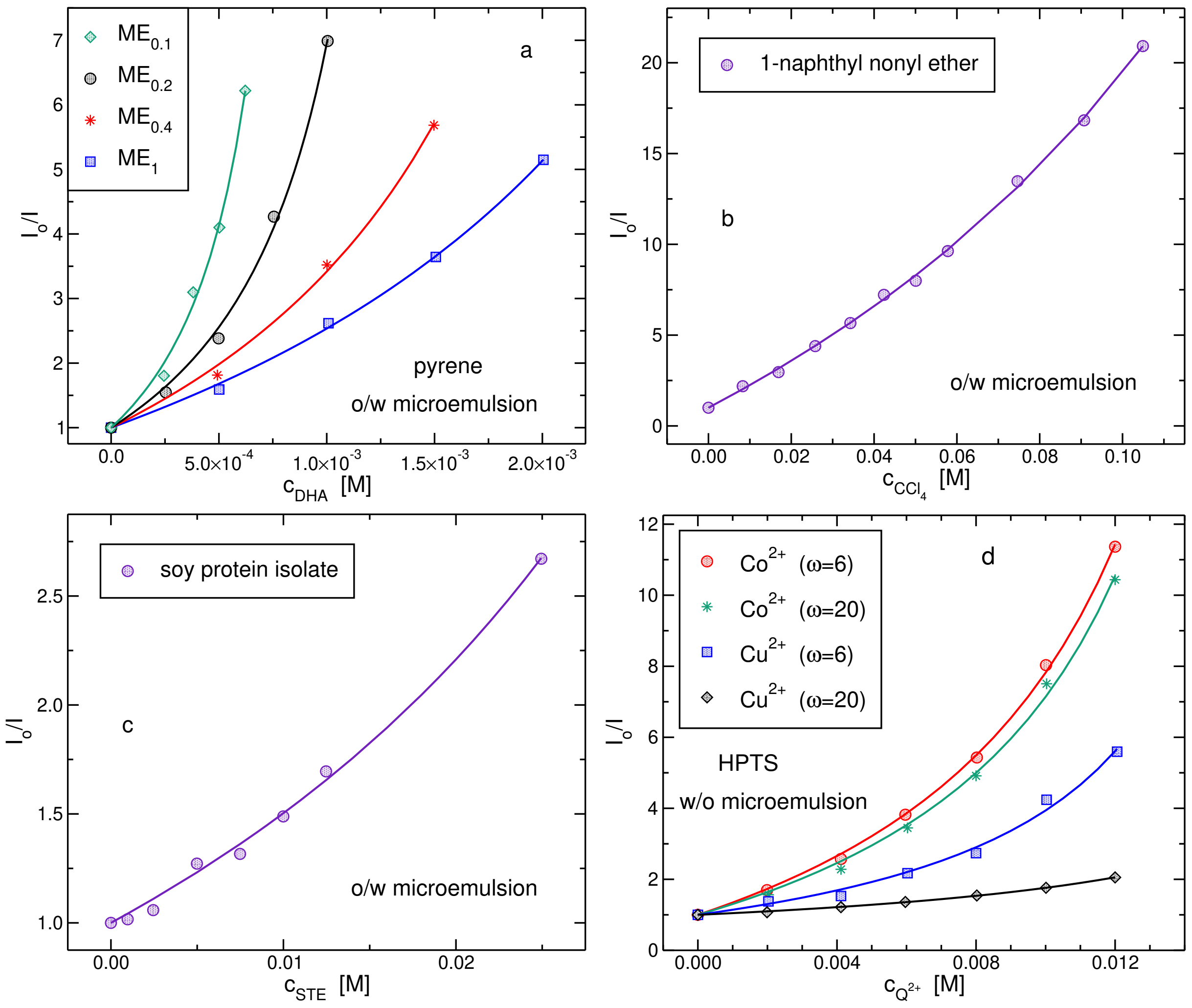

Figure S9.1: Fitting Eq. 44 to SV-plots of fluorescence in oil-in-water (o/w), or water-in-oil (w/o), microemulsions. Quenching of (a) pyrene by N,N-dihexylaniline, DHA, at different oil-to-water ratios, ME (Kano et al.[101]); (b) 1-naphthyl nonyl ether by carbon tetrachloride, CCl_4 (Panda et al.[102]); (c) soy protein isolate by stevioside, STE (Wan et al.[103]); and (d) 1-hydroxypyrene-3,6,8-trisulfonate (HPTS) by cobalt (II) and copper (II) cations at a two different water-to-oil ratios, ω (Biswas et al.[104]). Results of the fittings are given in Table S9.1.

Table S9.1: Results of Fitting Eq. 44 to fluorescence quenching of fluorophores confined inside microemulsions (Fig. S9.1).

Fluorophore	Quencher	plotted in	K	$\mathcal{Z}$	R
pyrene ($ME_{0.1}$)[101]	DHA	Fig. S9.1a	2040	1.50	0.9977
pyrene ($ME_{0.2}$)[101]	DHA	Fig. S9.1a	1460	1.44	0.9988
pyrene ($ME_{0.4}$)[101]	DHA	Fig. S9.1a	1340	1.24	0.9988
pyrene (ME_1)[101]	DHA	Fig. S9.1a	1020	1.20	0.9994
1-naphthyl nonyl ether[102]	CCl_4	Fig. S9.1b	117	1.03	0.9996
soy protein isolate[103]	stevioside	Fig. S9.1c	28.8	1.50	0.9981
HPTS ($\omega = 6$)[104]	Co^{2+}	Fig. S9.1d	276	1.19	0.9996
HPTS ($\omega = 20$)[104]	Co^{2+}	Fig. S9.1d	233	1.23	0.9988
HPTS ($\omega = 6$)[104]	Cu^{2+}	Fig. S9.1d	80.8	1.67	0.9956
HPTS ($\omega = 20$)[104]	Cu^{2+}	Fig. S9.1d	4.5	9.96	0.9996

SI-10 Fluorescence of Proteins

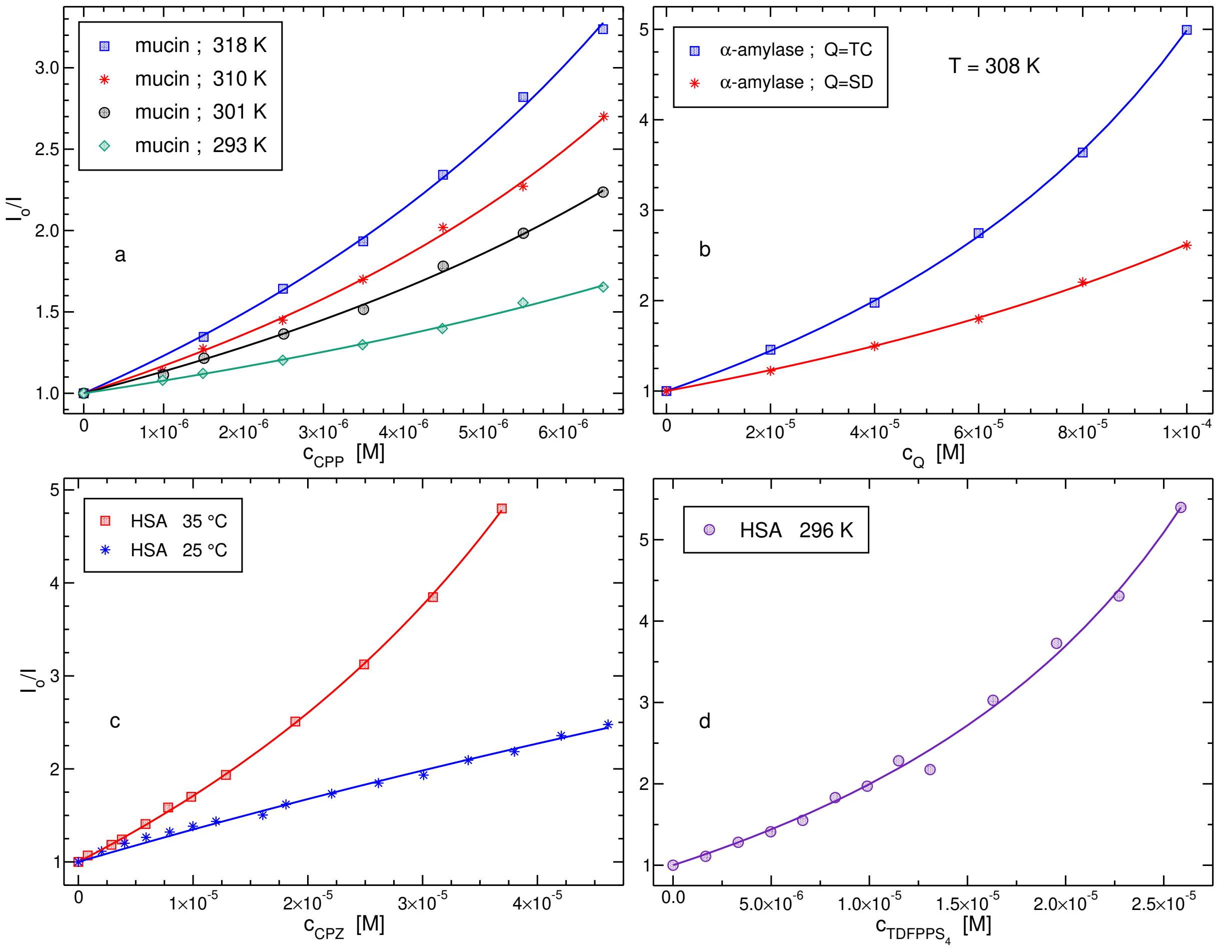

Figure S10.1: Fitting Eq. 44 to fluorescence quenching of tryptophan residues of various proteins. (a) Quenching of mucin (glycoprotein) by citrus peel pectin, CPP (Ahmad et al.[105]); (b) Quenching of α-amylase (hydrolytic enzyme) by tetracycline hydrochloride, TC, and by sulfadiazine, SD (Zhang et al.[33]); Quenching of human serum albumin, HSA, by (c) chlorpromazine, CPZ (Silva et al.[106]), and (d) 5,10,15,20-tetrakis(2,6-difluoro-3-sulfophenyl)porphyrin, $TDFPPS_4$ (Costa-Tuna et al.[107]); Results of the fittings are given in Table S10.1.

Table S10.1: Results of Fitting Eq. 44 to quenching of tryptophan residues of different proteins (Fig. S10.1).

Fluorophore	Quencher	plotted in	K	$\mathcal{Z}$	R
mucin; 318 K[105]	CPP	Fig. S10.1a	$1.59 \cdot 10^5$	1.37	0.9983
mucin; 310 K[105]	CPP	Fig. S10.1a	$9.90 \cdot 10^4$	1.60	0.9993
mucin; 301 K[105]	CPP	Fig. S10.1a	$7.65 \cdot 10^4$	1.67	0.9991
mucin; 293 K[105]	CPP	Fig. S10.1a	$3.26 \cdot 10^4$	2.28	0.9987
α-amylase; 308 K[33]	TC	Fig. S10.1b	$1.50 \cdot 10^4$	1.33	0.9999
α-amylase; 308 K[33]	SD	Fig. S10.1b	$7.48 \cdot 10^3$	1.45	0.9997
Human Serum Albumin; 35 °C[106]	CPZ	Fig. S10.1c	$5.35 \cdot 10^4$	1.19	0.9999
Human Serum Albumin; 25 °C[106]	CPZ	Fig. S10.1c	$3.93 \cdot 10^4$	0.916	0.9975
Human Serum Albumin; 296 K[107]	$TDFPPS_4$	Fig. S10.1c	$5.84 \cdot 10^4$	1.35	0.9976

SI-11 Homogeneous Solutions

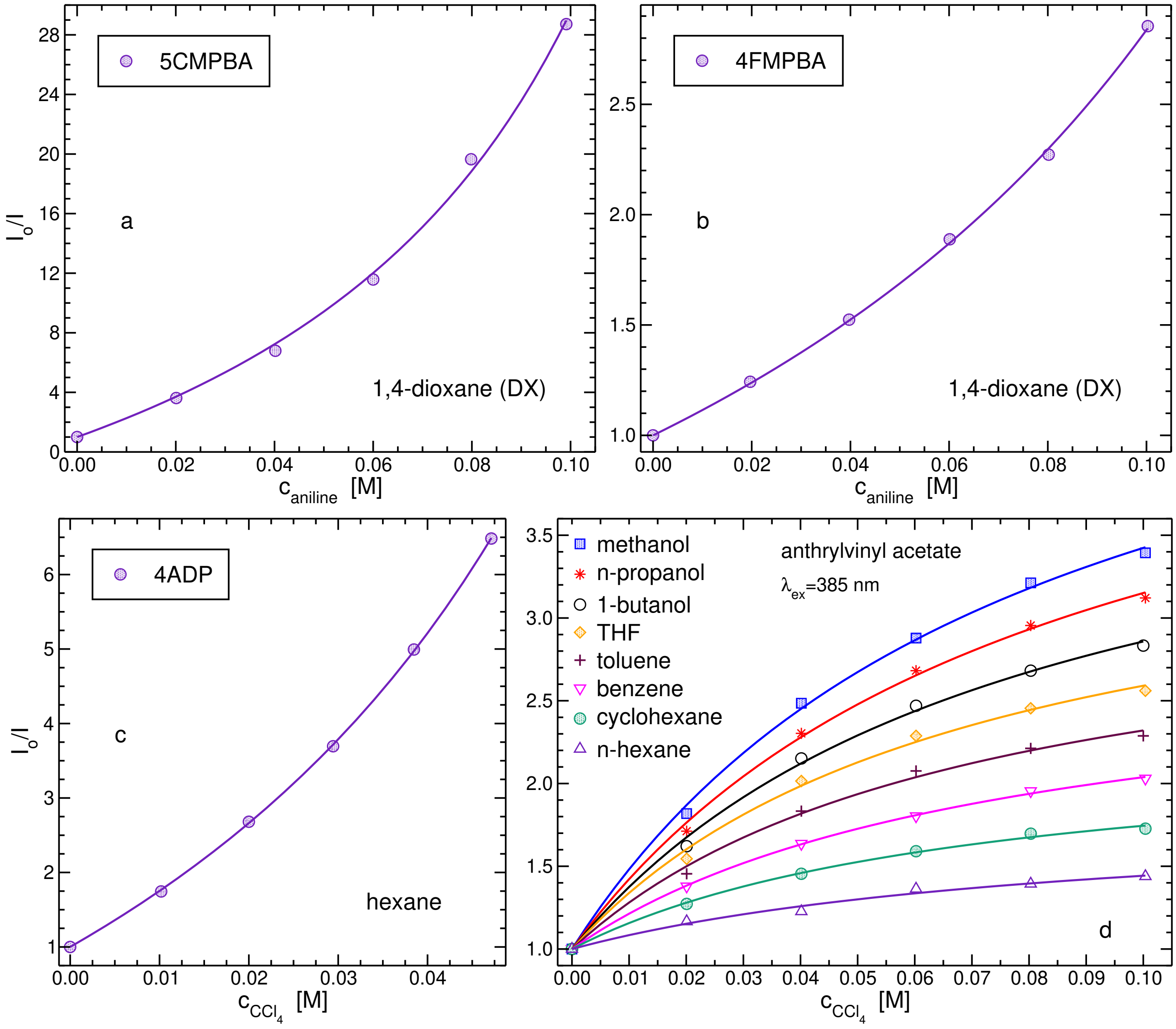

Figure S11.1: Fitting Eq. 44 to fluorescence quenching in homogeneous organic solvents. (a) Quenching of 5-chloro-2-methoxyphenylboronic acid, 5CMPBA, and (b) 4-fluoro-2-methoxyphenyl boronic acid, 4FMPBA, by aniline in 1,4-dioxane (Geethanjali et al.[108]); (c) Quenching of 4-aminodiphenyl, 4ADP, by tetrachloromethane in hexane (Swaminathan and Radha[109]); (d) Quenching of anthrylvinyl acetate, at $\lambda_{ex} = 385\ nm$, by tetrachloromethane in different organic solvents: methanol, n-propanol, 1-butanol, tetrahydrofuran (THF), toluene, benzene, cyclohexane, and n-hexane (Naik et al.[110]). Results of the fittings are given in Table S11.1.

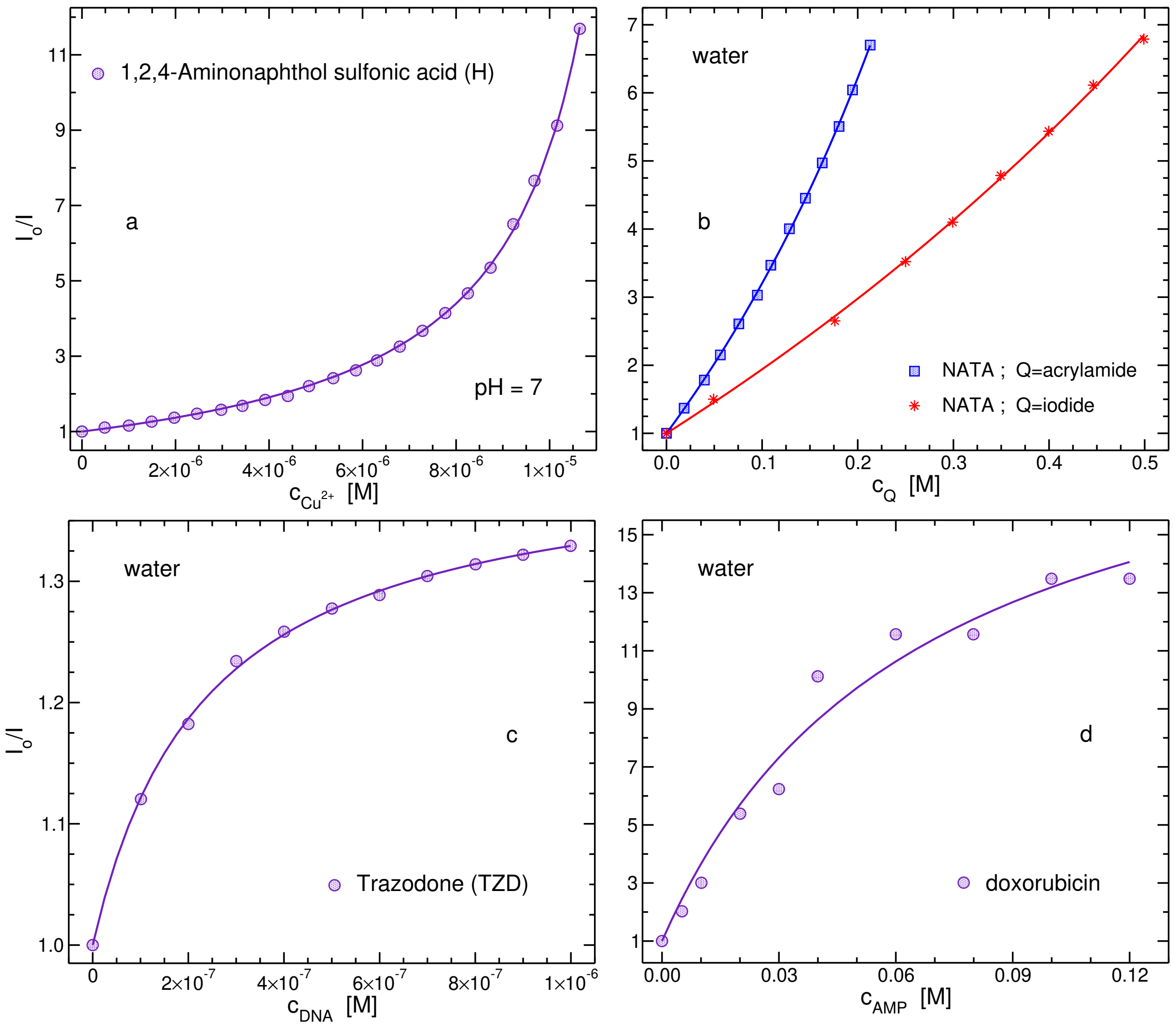

Figure S11.2: Fitting Eq. 44 to fluorescence quenching in homogeneous aqueous solutions. Quenching of (a) 1,2,4-Aminonaphthol sulfonic acid by copper(II) cation (Shahbaz et al.[111]); (b) N-acetyltryptophanamide, NATA, by acrylamide and iodide (Peak et al.[48]); (c) trazodone, TZD, by DNA (Kumar et al.[112]); (d) doxorubicin by adenosine 5'-monophosphate, AMP (Htun[113]). Results of the fittings are given in Table S11.1.

Table S11.1: Results of Fitting Eq. 44 to quenching of fluorophores in homogeneous organic solvents (Fig. S11.1) and in aqueous solutions (Fig. S11.2). The entries of anthrylvinyl acetate (Fig. S11.1d) refer to excitations at $\lambda_{ex} = 385\ nm$. (aq) refers to aqueous solution.

Fluorophore	Quencher	plotted in	K	$\mathcal{Z}$	R
5CMPBA; (1,4-dioxane)[108]	aniline	Fig. S11.1a	114	1.05	0.9990
4FMPBA; (1,4-dioxane)[108]	aniline	Fig. S11.1b	6.99	1.57	0.9997
4ADP; (hexane)[109]	CCl_4	Fig. S11.1c	59.9	1.15	1.000
anthrylvinyl acetate; (methanol)[110]	CCl_4	Fig. S11.1d	66.4	0.815	0.9993
anthrylvinyl acetate; (n-propanol)[110]	CCl_4	Fig. S11.1d	59.2	0.798	0.9992
anthrylvinyl acetate; (1-butanol)[110]	CCl_4	Fig. S11.1d	55.0	0.768	0.9989
anthrylvinyl acetate; (THF)[110]	CCl_4	Fig. S11.1d	53.1	0.730	0.9982
anthrylvinyl acetate; (toluene)[110]	CCl_4	Fig. S11.1d	46.4	0.692	0.9981
anthrylvinyl acetate; (benzene)[110]	CCl_4	Fig. S11.1d	37.5	0.645	0.9997
anthrylvinyl acetate; (cyclohexane)[110]	CCl_4	Fig. S11.1d	31.9	0.561	0.9988
anthrylvinyl acetate; (n-hexane)[110]	CCl_4	Fig. S11.1d	20.3	0.458	0.9930
aminonaphthol sulfonic acid; (aq)[111]	Cu^{2+}	Fig. S11.2a	$7.48 \cdot 10^4$	2.06	0.9998
N-acetyltryptophanamide; (aq)[48]	acrylamide	Fig. S11.2b	17.7	1.08	0.9999
N-acetyltryptophanamide; (aq)[48]	iodide	Fig. S11.2b	8.49	1.06	0.9998
trazodone (TZD); (aq)[112]	DNA	Fig. S11.2c	$5.98 \cdot 10^6$	0.289	0.9996
doxorubicin; (aq)[113]	AMP	Fig. S11.2d	321	0.953	0.9869

SI-12 Derivation of Simultaneous Dynamic and Static Quenching of Single-Fluorophore Systems

Quenching of fluorescence by simultaneous operation of dynamic and static mechanisms is obviously more complicated to describe. To simplify the model, we assume, the concentration of ground-state complex (due to static mechanism) is much smaller than total quencher concentration, and that the contribution of dynamic quenching is not negligible. We then utilize the relation in Eq. 31

$$\frac{I_\circ}{I_Q} = \left[1 - \frac{k_i\langle c_{(FQ)^*}\rangle}{k_a\langle c_F\rangle_Q}\right]^{-1} = 1 + \frac{k_i\langle c_{(FQ)^*}\rangle}{k_a\langle c_F\rangle_Q - k_i\langle c_{(FQ)^*}\rangle} \qquad , \tag{S1}$$

where the concentration of unbound ground-state fluorophore, $\langle c_F\rangle_Q$, is a function of the binding constant of ground-state complex formation, K_{FQ}. For consistency with above derivations, we choose to describe $\langle c_F\rangle_Q$ as,

$$\langle c_F\rangle_Q = \langle c_F\rangle_{Q,d} - \langle c_{FQ}\rangle \qquad , \tag{S2}$$

where $\langle c_F\rangle_{Q,d}$ is the concentration in the absence of static mechanism, thus in pure dynamic quenching, and as noted before, considered to be independent on quencher's concentration (at least for the range of concentrations measured in fluorescence quenching experiment). We then substitute this expression of $\langle c_F\rangle_Q$ into Eq. S1 and obtain,

$$\frac{I_\circ}{I_Q} = 1 + \frac{k_i\langle c_{(FQ)^*}\rangle}{k_a\left(\langle c_F\rangle_{Q,d} - \langle c_{FQ}\rangle\right) - k_i\langle c_{(FQ)^*}\rangle} \qquad . \tag{S3}$$

Once again, assuming single-fluorophore (sub)systems we utilize the relations in Eq. 32 and Eq. 38 to express the ratio of fluorescence intensities as,

$$\begin{aligned}
\frac{I_\circ}{I_Q} &= 1 + \frac{k_i \frac{K_{(FQ)^*}\cdot c_Q^{\text{total}}}{V(c^{\varnothing} + K_{(FQ)^*}\cdot c_Q^{\text{total}})}}{k_a\langle c_F\rangle_{Q,d} - k_a\frac{K_{FQ}\cdot c_Q^{\text{total}}}{V(c^{\varnothing}+K_{FQ}\cdot c_Q^{\text{total}})} - k_i\frac{K_{(FQ)^*}\cdot c_Q^{\text{total}}}{V(c^{\varnothing}+K_{(FQ)^*}\cdot c_Q^{\text{total}})}} \\
&= 1 + \frac{\frac{k_i}{k_a\langle N_F\rangle_{Q,d}}K_{(FQ)^*}\cdot c_Q^{\text{total}}}{c^{\varnothing} - \frac{1}{\langle N_F\rangle_{Q,d}}\left[\frac{c^{\varnothing}+K_{(FQ)^*}\cdot c_Q^{\text{total}}}{c^{\varnothing}+K_{FQ}\cdot c_Q^{\text{total}}}\right]K_{FQ}\cdot c_Q^{\text{total}} + \left(1 - \frac{k_i}{k_a\langle N_F\rangle_{Q,d}}\right)K_{(FQ)^*}\cdot c_Q^{\text{total}}} \\
&= 1 + \frac{\mathcal{Z}_d K_{(FQ)^*}\cdot c_Q^{\text{total}}}{c^{\varnothing} + (1-\mathcal{Z}_d)\,K_{(FQ)^*}\cdot c_Q^{\text{total}} - \frac{1}{\langle N_F\rangle_{Q,d}}\left[\frac{c^{\varnothing}+K_{(FQ)^*}\cdot c_Q^{\text{total}}}{c^{\varnothing}+K_{FQ}\cdot c_Q^{\text{total}}}\right]K_{FQ}\cdot c_Q^{\text{total}}} \qquad ,
\end{aligned} \tag{S4}$$

where $\mathscr{Z}_d$ is defined in Eq. 34. When static quenching is negligible $(K_{FQ} \to 0)$, the last term in the denominator after the last equality vanishes and Eq. S4 reduces to the dynamic quenching equation (Eq. 35).

SI-13 Comparison of $\mathcal{Z}K$ with K_{SV} at low c_Q^{total}

Table S13.1: Comparisons between the value of $\mathcal{Z}K/c^{\varnothing}$ obtained from fitting the non-linear SV-plot to Eq. 44 (considering the entire reported range of quencher's concentration) and the value of K_{SV}, determined by the authors when considering only the linear segment of the curve observed at low quencher's concentrations, K_{SV}^{lc}.

Fluorophore	Quencher	plotted in	K_{SV}^{lc} (M^{-1})	$\mathcal{Z}K/c^{\varnothing}$ (M^{-1})
Terbium-based MOF[61]	thiamethoxam	Fig. S1.2a	$3.441 \cdot 10^4$	$2.82 \cdot 10^4$
Eu^{3+} (doped SrF_2 PEG-NP)[63]	ADP	Fig. S1.2d	4.91	6.51
OFX $293\ ^\circ C$ (β-cyclodextrin)[64]	Cu^{2+}	Fig. S2.1a	$1.77 \cdot 10^5$	$1.32 \cdot 10^5$
OFX $303\ ^\circ C$ (β-cyclodextrin)[64]	Cu^{2+}	Fig. S2.1a	$1.58 \cdot 10^5$	$1.12 \cdot 10^5$
OFX $313\ ^\circ C$ (β-cyclodextrin)[64]	Cu^{2+}	Fig. S2.1a	$1.37 \cdot 10^5$	$1.00 \cdot 10^5$
allyloxy-based MOF[66]	Pd^{2+}	Fig. S2.2a	$1.05 \cdot 10^5$	$5.18 \cdot 10^3$
C151 (SDS)[76]	DMAN	Fig. S4.2a	13.4	10.9
C153 (SDS)[76]	DMAN	Fig. S4.2a	5.70	6.44
C481 (SDS)[76]	DMAN	Fig. S4.2a	4.86	4.03
DHAQ (SDS)[78]	DMPT	Fig. S4.2d	14.472	14.2
PTE-BS/TBA[86]	MV^{2+}	Fig. S6.1b	$1.4 \cdot 10^4$	$1.66 \cdot 10^4$
MBL-PPV (TBA)[43]	MV^{2+}	Fig. S6.1d	$1.9 \cdot 10^7$	$2.09 \cdot 10^7$
TPE containing PATA (PI)[88]	picric acid	Fig. S6.2a	$2.1 \cdot 10^4$	$1.98 \cdot 10^4$
HSP-TPE copolymer[89]	picric acid	Fig. S6.2b	$2.4 \cdot 10^4$	$1.43 \cdot 10^4$
HTPEAIM[90]	picric acid	Fig. S6.2c	$2.67 \cdot 10^4$	$1.67 \cdot 10^4$

Table S13.2: Continuation of Table S13.1.

Fluorophore	Quencher	plotted in	K_{SV}^{lc} (M^{-1})	$\mathcal{Z}K/c^{\varnothing}$ (M^{-1})
Ur-PMMA-b-PVAc[91]	picric acid	Fig. S6.2d	$1.506 \cdot 10^4$	$1.35 \cdot 10^4$
Ur-PMMA-b-PVAc[91]	nitrotoluene	Fig. S6.2e	$1.422 \cdot 10^4$	$2.22 \cdot 10^4$
Ur-PMMA-b-PVAc[91]	nitrophenol	Fig. S6.2e	$1.147 \cdot 10^4$	$1.58 \cdot 10^4$
Ur-PMMA-b-PVAc[91]	nitrobenzene	Fig. S6.2e	$1.037 \cdot 10^4$	$1.81 \cdot 10^4$
Ur-PMMA-b-PVAc[91]	p-nitroaniline	Fig. S6.2e	$8.38 \cdot 10^3$	$1.59 \cdot 10^3$
Ur-PMMA-b-PVAc[91]	Fe^{3+}	Fig. S6.2f	$2.64 \cdot 10^3$	$2.31 \cdot 10^4$
Ur-PMMA-b-PVAc[91]	Cu^{2+}	Fig. S6.2f	$4.82 \cdot 10^3$	$5.74 \cdot 10^3$
Ur-PMMA-b-PVAc[91]	$UO_2{}^{2+}$	Fig. S6.2f	$2.99 \cdot 10^3$	$2.22 \cdot 10^3$
1-naphthyl nonyl ether[102]	CCl_4	Fig. S9.1b	98.55	121
Human Serum Albumin; 35 °C[106]	CPZ	Fig. S10.1c	$6.7 \cdot 10^4$	$6.37 \cdot 10^4$
Human Serum Albumin; 25 °C[106]	CPZ	Fig. S10.1c	$4.1 \cdot 10^4$	$3.60 \cdot 10^4$
